# Novel microscopy-based screening method reveals regulators of contact-dependent intercellular transfer

**DOI:** 10.1038/srep12879

**Published:** 2015-08-14

**Authors:** Dominik Michael Frei, Erlend Hodneland, Ivan Rios-Mondragon, Anne Burtey, Beate Neumann, Jutta Bulkescher, Julia Schölermann, Rainer Pepperkok, Hans-Hermann Gerdes, Tanja Kögel

**Affiliations:** 1 Department of Biomedicine, University of Bergen, Jonas Lies Vei 91, N-5009 Bergen, Norway; 2 Advanced Light Microscopy Facility, European Laboratory of Molecular Biology (EMBL), Meyerhofstraße 1, 69117 Heidelberg, Germany

## Abstract

Contact-dependent intercellular transfer (codeIT) of cellular constituents can have functional consequences for recipient cells, such as enhanced survival and drug resistance. Pathogenic viruses, prions and bacteria can also utilize this mechanism to spread to adjacent cells and potentially evade immune detection. However, little is known about the molecular mechanism underlying this intercellular transfer process. Here, we present a novel microscopy-based screening method to identify regulators and cargo of codeIT. Single donor cells, carrying fluorescently labelled endocytic organelles or proteins, are co-cultured with excess acceptor cells. CodeIT is quantified by confocal microscopy and image analysis in 3D, preserving spatial information. An siRNA-based screening using this method revealed the involvement of several myosins and small GTPases as codeIT regulators. Our data indicates that cellular protrusions and tubular recycling endosomes are important for codeIT. We automated image acquisition and analysis to facilitate large-scale chemical and genetic screening efforts to identify key regulators of codeIT.

Intercellular communication is indispensable for development, homeostasis, repair and immune defence in multicellular organisms. Dysregulation of intercellular communication is a hallmark of pathological conditions such as cancer and autoimmune diseases, and pathogens can hijack the communication. Exchange of material between cells can occur without direct cell-to-cell contact by release of macromolecular complexes and membrane-enveloped organelles, such as microvesicles and exosomes, into the extracellular environment. Cell-to-cell contact dependent communication through gap junctions allows exchange of small molecules. Large molecules and organelles are also transferred between cells in contact-dependent processes, for example melanosomes from melanocytes to keratinocytes in the skin^1^,^2^. Another example is the exchange of membrane at the immunological synapse, termed trogocytosis (for a review see^3^). We propose the term contact-dependent intercellular transfer (denoted “codeIT”) to distinguish these forms of intercellular transfer from contact-independent transfer. The most frequently described structures mediating codeIT in non-immune cells are nanotubes (also called tunnelling nanotubes).

Nanotubes are 70–800 nm thin membranous structures, which connect cells and can be several cell diameters long^4^. They contain filamentous actin (F-actin), and are ubiquitous in cultured cells. Importantly, nanotubes have also been reported in embryos and tissue (for review see^5^). The cargos reported to be transported by nanotube-mediated codeIT include membrane-associated proteins^6^,^7^, endosomes^8^, lysosomes^9^, mitochondria^10^,^11^, and RNA^12^,^13^, amongst them tumor-promoting factors^6^,^7^,^10^,^14^. CodeIT can result in physiologically relevant reactions, including phosphorylation of ERK^15^, down-regulation of SMAD signalling^16^, changes in cell proliferation^7^,^12^, inhibition of apoptosis^9^,^11^ and cancer drug resistance^6^,^10^. At the immunological synapse, codeIT plays an important role in antigen presentation via trogocytosis^17^,^18^,^19^ and regulates NK cell cytotoxicity^20^. Various pathogens have been shown to spread in a contact-dependent way, including prions^21^, viruses^22^,^23^,^24^ and bacteria^25^,^26^,^27^,^28^,^29^,^30^.

Despite the physiological relevance of codeIT, the molecular mechanism remains elusive, and specific molecular markers for both nanotubes and codeIT are lacking. One reason for this is the fragility of nanotubes. Nanotubes break upon fixation and light exposure, which makes their investigation challenging. Until now a sufficiently specific and reproducible quantification method of codeIT has been lacking. Previous approaches employed donor cells carrying a transferrable fluorescent label which were co-cultured with counterstained acceptor cells at a 1:1 ratio and quantification of transfer by either flow cytometry^13^,^31^ or manual evaluation of micrographs^8^,^21^. Typically, cells were seeded in relatively low density to visualize nanotubes in parallel. However, fluctuating baseline signals, low signal-to-noise ratio and insufficient exclusion of contact-independent transfer made these methods unsuitable for screening. In order to develop a robust screening protocol for codeIT markers, we focused on the intercellular transfer of organelles stained with the lipophilic dye Vybrant® DiD (DiD)^4^,^8^,^32^, a phenomenon which has also been observed *in vivo*^33^. Inhibition with cytochalasins has shown that DiD transfer is dependent on F-actin^8^,^32^. We developed a new quantification method for codeIT, based on an improved co-culture system, confocal microscopy, and automated image processing and analysis. This method was applied in an siRNA screen and subsequent hit validation. To demonstrate the relevance of the method and our findings for cancer research, we inhibited the transfer of oncogenic H-Ras by disrupting actin polymerisation with Cytochalasin D or by knockdown of CDC42. Finally, in order to enable larger scale screening approaches, we automated pattern recognition of region(s) of interest (ROI(s)) and image acquisition.

## Results

### Confluent co-cultures with low donor-to-acceptor ratio allow codeIT quantification

**Figure 1:**
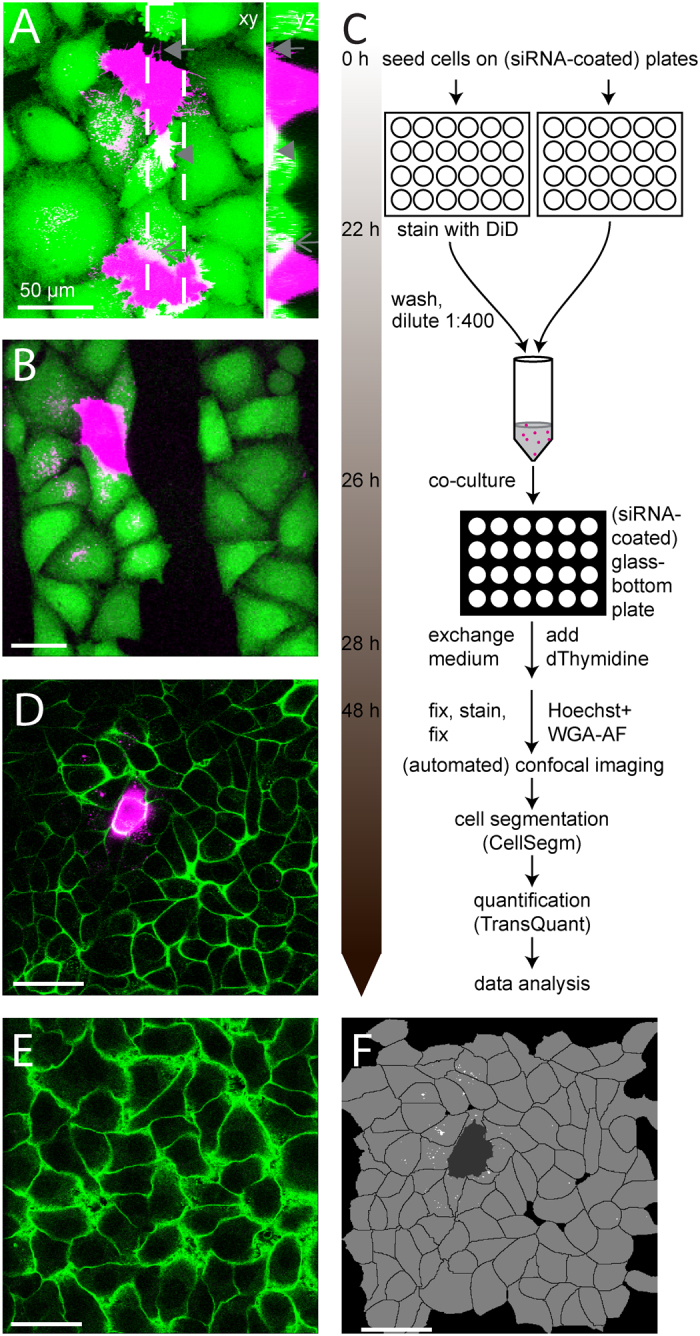
CodeIT can be assigned to individual donor cells, quantified and inhibited. Cells were separately labelled with DiD (magenta) and CTG (**A** and **B**; green) or not labelled, then mixed with a low percentage of DiD cells, co-cultured for 22 h, fixed, and imaged by confocal microscopy. Co-cultures with non-labelled cells were stained with WGA-AF-488 after fixation (**D** and **D’**; green). For details see methods. Shown are maximum projections of DiD and CTG channels (**A**–**B**) and a maximum projection of the DiD channel and a single confocal plane 2 μm above the substrate in the WGA-AF-488 channel (**D** and **E**). (**A**) Diverse contact-structures in confluent cultures: nanotube-like (arrow), flat protrusions underneath adjacent cells (arrowhead) and on top (arrow). Note that codeIT of DiD occurs close to these sites and that in order to visualize transfer, donor cells had to be overexposed. (**B**) DiD transfer is contact-dependent. Cells were plated on a substrate containing stripes rendered non-adhesive for cells (see methods). Note that transfer does not cross the resulting gap between cells, even though the gap was smaller than the extension of the transfer pattern. (**C**) Workflow diagram of quantification method. (**D**) Areas imaged contained either one DiD-labelled cell (**D**), or none (**D’**) as background, with no further DiD cell within 200 μm. DiD transfer can therefore be assigned to its donor cell of origin. (**E**) CodeIT is quantified by TransQuant software based on cell segmentation by CellSegm software using the WGA-AF cell surface signal. Shown is the result of cell segmentation by CellSegm of the field of view shown in D for a single plane. Acceptor cells, shown in light grey and non-cell area shown in black were segmented in 3D. TransQuant automatically recognises the DiD-labelled donor cell (dark grey) and quantifies codeIT in the acceptor cells (white). Scale bars: 50 μm.

In order to achieve sufficient amounts of transfer for screening, we needed to increase the amount of transfer compared to previous approaches. We reasoned that with increasing culture density, intercellular contact increases and thereby probably also the amount of contact-dependent transfer. Indeed, intercellular DiD transfer increased with higher culture density, up to confluence. We observed intercellular contact via several distinct types of structures, including nanotube-like connections and thicker protrusions underneath and on top of adjacent cells, and that codeIT of DiD occurred close to these sites (Fig. 1A). However, in confluent co-cultures with equal numbers of DiD-labelled donor cells and unlabelled acceptor cells, the ratio between codeIT and transfer through conditioned medium varied widely between experiments, with no reproducible dominance of one factor over the other. Taken together, codeIT was indistinguishable from transfer through the medium (Supplementary Fig. S1 online).

To circumvent this problem, we used a low ratio of DiD-stained donor to unstained acceptor cells in confluent culture, resulting in a reduction of the contribution of transfer through the medium. For better signal-exploitation and specificity, we evaluated codeIT microscopically, as described in the following. The use of the slowly migrating HeLa-Kyoto cell line confined DiD transfer to a restricted field. To demonstrate the dominance of contact-dependent over -independent transfer in our optimised co-culture protocol, we introduced intercellular gaps into our cell cultures. Therefore, alternating cell adhesive and non-adhesive stripes were stamped onto the substrate, thus restricting attachment of cells to adhesive stripes and preventing formation of protrusions spanning the non-adhesive gaps. This restricted the transfer of DiD in low-ratio (1:100) co-cultures to cells on the same side of the gap as the donor cell (Fig. 1B). Hence, DiD is a specific marker for codeIT in this configuration.

**Figure 2:**
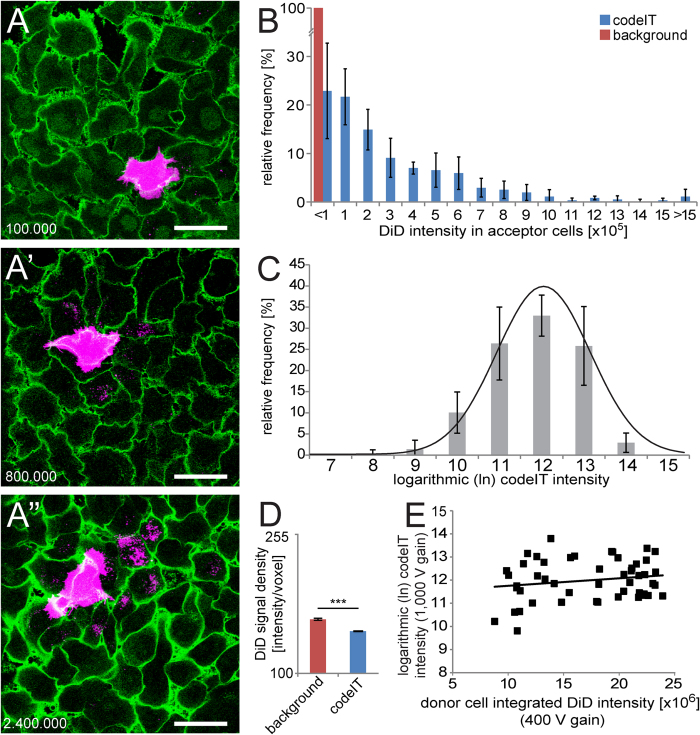
Distribution of codeIT intensity and its relation to donor cell DiD intensity. (**A**–**A**”) Images display representative examples of DiD signal for low (**A**), medium (**A’**) and high (**A**”) codeIT intensity. Maximum projections of DiD above threshold (magenta); single confocal plane 2 μm above substrate level of WGA-AF-488 (green); the corresponding integrated codeIT intensity is indicated by white numbers in the lower left corner of each image; scale bars, 50 μm. (**B**) Histogram of integrated codeIT (DiD) intensities. Transfer below background, <100 000; transfer above background, 100 000 to >1 500 000. N = 825 stacks of 8 independent experiments treated with control siRNA; bars, mean; error bars, SD. (**C**) Histogram of data shown in (**B**) after logarithmic (base e) transformation. Note normal distribution for DiD transfer intensity. Test for normality: Chi-square test, p = 0.934. (**D**) DiD signal density (intensity/voxel) compared between background and codeIT. ***two-sided independent Student’s t-test p < 10^−40^. (**E**) Integrated DiD transfer intensity (detector set to 1,000 V) plotted against donor cell integrated DiD intensity (detector set to 400 V). DiD intensity measured in same stacks as transfer but with lower detector voltage to avoid overexposing donor cells. Note independence of the two parameters. Line: linear regression. N = 51 stacks pooled from 3 independent experiments treated with control siRNA; R^2^ = 0.003; Pearson’s correlation index = 0.05 indicating no correlation of donor cell and transfer intensity within the investigated range.

In our final screening protocol (Fig. 1C), we first cultured equal amounts of cells in two separate wells for 24 h. For knockdown experiments, these wells were coated with siRNA. Thereafter, the cells in one well were stained with DiD to be used as codeIT donor cells. Subsequently, we diluted the donor cells and mixed them with unlabelled acceptor cells at a ratio of 1 donor per 400 acceptor cells. We chose dilution over sparser cultures to improve reproducibility and in order to keep culture conditions for donor and acceptor cells identical. The mix was then co-cultured on a glass-bottom plate for 22 h prior to fixation, nuclear and plasma membrane (PM) staining, and microscopy and image analysis. Only regions containing single donor cells in a confluent lawn of acceptor cells were imaged to measure codeIT (Fig. 1D), while background, which includes transfer through the medium, was measured in images of adjacent areas that contain only acceptor cells and not donor cells (Fig. 1E). Both types of areas, those for analysing background and those for analysing codeIT, were required to be at least 200 μm distant from any other donor cells. This prevented overlap of transfer patterns from multiple donor cells and as a result, codeIT could be attributed to specific donor cells. Doublets and groups of donor cells were excluded from the analysis, as well as small, rounded and partially detached donor cells, as they were assumed to be dying. Multi-nucleated donor cells were also excluded. Membrane staining with wheat-germ-agglutinin coupled to Alexa-Fluor-488 (WGA-AF-488) allowed automatic cell segmentation using CellSegm^34^. Subsequently, we quantified the DiD intensity in each cell by TransQuant, a software we programmed for this purpose (https://github.com/dominikfr/Transquant). This software recognises donor cells based on their high DiD intensity and control areas by the lack of a donor cell. The volume of the donor cell, based on the DiD signal and watershed volume from CellSegm, was dilated by one voxel to avoid falsely quantifying parts of the donor cell as transfer. DiD signal located inside acceptor cells was found based on the cell volumes defined by the cell segmentation. A threshold was set to eliminate signal due to autofluorescence, above which signal was considered to be intercellular transfer (Fig. 1F). Acceptor cells with a very small volume were automatically excluded by the software in order to avoid measuring increased auto-fluorescence of dead or dying cells as transfer. The results for each experiment were exported as a text file, easily readable by common software. The results showed, at 22 h of co-culture, transfer intensity varied widely, with more cells with low amounts of transfer and few donor cells with high amounts of transfer (Fig. 2A,B). A log-transformation of the data resulted in normal distribution (Fig. 2C). The majority (>77%) of donor cells had transferred DiD above background (Fig. 2B). Transfer signal measured by this method differed not only from the background in amount, but also in density (Fig. 2D), suggesting different origins for the bulk of signal measured in background areas versus donor cell areas. The integrated signal intensity of the transfer originating from single donor cells did not correlate with the staining intensity of the respective donor cells (Fig. 2E). For the purpose of this quantification, the staining intensity of the donor cells was acquired with a lower detector voltage, resulting in images without large saturated areas.

### Live-cell imaging of codeIT

Live-cell imaging experiments confirmed that acceptor cells in direct contact with donor cells acquired DiD signal over time, even if a constant laminar flow of medium was applied in a microfluidic channel, restricting lateral diffusion through the medium and clearing any compounds released from the cells into the medium swiftly. The transferred material rearranges under cell division (Movie 1). Notably, DiD-stained protrusion bundles from donor cells oriented dynamically towards the bulk of transferred material, which localised to the perinuclear region in the acceptor cells (Movie 2).

In confocal 3D time-lapse, we followed the transfer of vesicles from the bulk donor cell signal into acceptor cells, where the vesicle moved towards the bulk of transferred DiD vesicles and stayed there (Movie 3). The observed transfer of DiD-stained structures in live-cell imaging and the dotted distribution in the acceptor cells are consistent with a transfer of DiD in larger complexes rather than diffusion of individual molecules.

### CodeIT is dependent on F-actin and serum components

**Figure 3:**
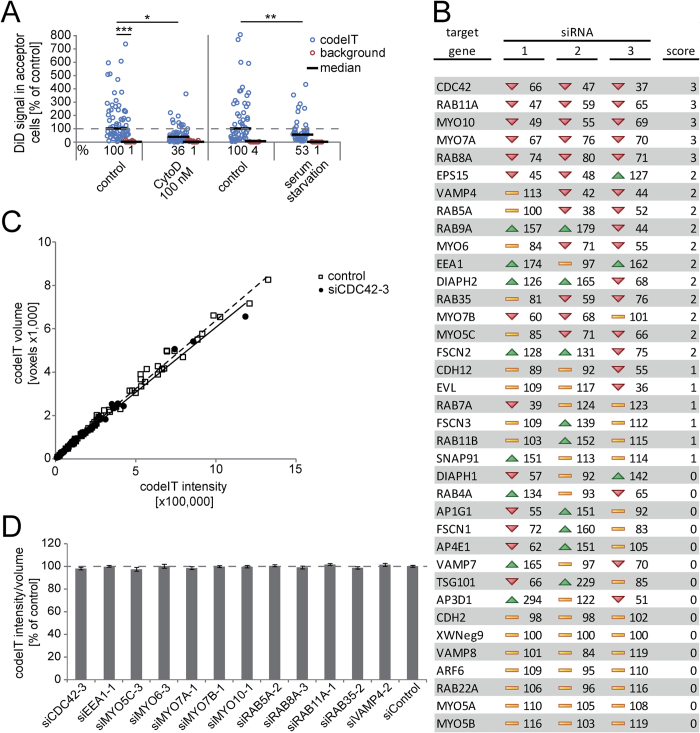
Effects of CytoD, serum starvation and siRNA-mediated knockdown on codeIT of DiD. (**A**) Cells were treated for 22 h in four different conditions: DMSO as a control, 100 nM cytochalasin D dissolved in DMSO, not starved of serum as a control or starved of serum. CodeIT was quantified automatically by our method. In short, codeIT was measured as the sum of DiD signal intensity in all acceptor cells within the ROI for each donor cell. Data is depicted as dot plots with each dot representing one analysed donor cell. Median values for each condition are represented by black bars. Background levels were measured in cells at > 200 μm distance from any donor cell. N ≥ 46 for transfer, ≥9 for background, 3 independent experiments. *ANOVA, followed by *post hoc* Dunnett’s test, p < 0.05; **2-sided independent Student’s t-test, p < 0.005; ***2-sided independent Student’s t-test p < 10^−19^. (**B**) Summary of siRNA screening results. Median codeIT intensity of cells expressing candidate siRNA compared to control siRNA (in % of control siRNA) shown for all three different siRNAs per target gene. Scores (column 5) were assigned to the following criteria: Number of siRNAs targeting the same gene that showed effects with similar direction. The density of codeIT was measured as the ratio of integrated DiD intensity per number of voxels, above threshold intensity, for cells transfected with either control siRNA or siRNAs that affected DiD transfer intensity. (**C**) The integrated intensity of codeIT (sum of voxels multiplied with greyscale units) was plotted against the integrated volume of codeIT (sum of voxels) originating from two different conditions: Individual donor cells for control (open boxes) and cells treated with si*CDC42*-3, which had reduced codeIT intensity to 37% of control (filled circles). Note that volume and intensity of codeIT correlate linearly in both conditions. R^2^ = 0.991 (control) and 0.989 (si*CDC42*-3). Pearson’s correlation index = 0.984 (control) and 0.974 (si*CDC42*-3). (D) DiD intensity per volume for siRNAs with strong effects on codeIT. Note that the ratio is constant for all siRNAs. No significant differences in the intensity/volume ratios were observed (ANOVA).

F-actin and serum dependency of codeIT was tested by treating co-cultures with 100 nM cytochalasin D (CytoD) or serum starvation for 22 h. Both CytoD treatment, and serum starvation significantly inhibited the transfer of DiD in comparison to controls (Fig. 3A). We concluded that both F-actin and serum components are necessary for codeIT.

### SiRNA screen identifies codeIT regulating genes

In order to investigate the mechanism underlying codeIT, we performed a pilot screening with siRNAs targeting 36 selected genes. For each target, three different siRNA sequences were transfected separately, each in three independent experiments, and compared to non-targeting control siRNA. Transfection with siRNAs was carried out using a reverse transfection protocol, *i.e.* plating cells on top of an siRNA-coated surface, as described previously^35^. For a list of all siRNAs used in the screening, see Supplementary Table S1 online. All cells were transfected twice: first, cells were plated onto siRNA coated plates 24 h before co-culture, and then co-cultured on plates coated with siRNAs identical to those in the first transfection step. The reason for this was that single transfection of siRNA for only the first 24 h was generally less efficient. After 22 h of co-culture, fixation, staining and imaging, codeIT was quantified by TransQuant after cell segmentation by CellSegm (see methods). Several siRNAs clearly affected the amount of codeIT (Fig. 3B). Notably, only the volume, *i.e.* the number of voxels, but not the mean ratio of intensity/voxel of the codeIT signal deviated from the control value (*e.g.* siCDC42-3, which had the strongest effect in the screen; Fig. 3C). This was the case for all hit siRNAs (shown for the siRNAs with the strongest effects on codeIT, Fig. 3D).

### Candidate gene validation by overexpression of EGFP-tagged proteins

**Figure 4:**
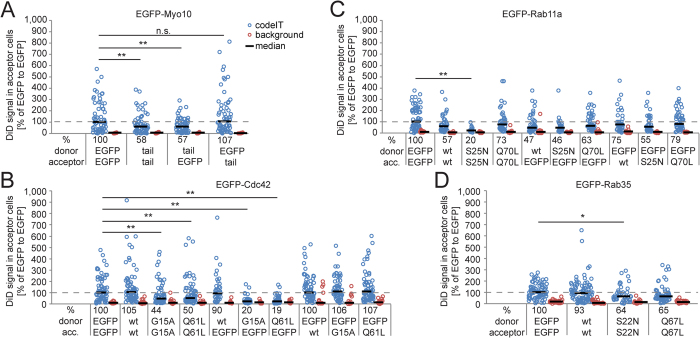
Validation of siRNA screening hits by overexpression of EGFP-tagged proteins. Cells stably expressing EGFP, EGFP-Myo10-tail (tail), EGFP-Rab11a (wt), EGFP-Rab11-S25N (S25N), EGFP-Rab11-Q70L (Q70L), EGFP-Cdc42 (wt), EGFP-Cdc42-G15A (G15A), EGFP-Cdc42-Q61L (Q61L), EGFP-Rab35 (wt), EGFP-Rab35-S22N (S22N), EGFP-Rab35-Q67L (Q67L) were established and co-cultured at low donor/acceptor cell ratio as donor and acceptor cells or either one. CodeIT (blue circles) and background (red circles) represent integrated DiD signal in acceptor cells. Transfer of DiD in co-cultures of DiD-stained EGFP-expressing cells as donors and unstained EGFP-expressing cells as acceptors was set to 100%. (**A**) EGFP-Myo10-tail significantly reduces codeIT when expressed in donor and acceptor cells or only donor cells, but not when expressed only in acceptor cells. (**B**) EGFP-Cdc42-wild-type expression had no significant effect on codeIT, while both, G15A and Q61L mutants, significantly reduced codeIT when expressed in donor and acceptor cells or only donor cells but not when expressed only in acceptor cells. (**C**) EGFP-Rab11a-wild-type, -S25N and -Q70L significantly reduced codeIT when expressed in donor and acceptor cells. Changes upon expression in only acceptor or only donor cells were not statistically significant. Effect of EGFP-Rab11a-wild-type and -Q70L expression in donor and acceptor compared to expression in both donor and acceptor cells are consistent with the sum of reductions in donors and acceptors, while the effect of -S25N is consistent with a synergistic reduction in donors and acceptors. (**D**) Stable expression of EGFP-Rab35-wild-type did not affect codeIT, while -S22N and -Q67L mutants both significantly reduced codeIT when expressed in donor and acceptor cells. Significance was tested by ANOVA, followed by *post-hoc* Dunnett’s test. *p < 0.05, **p < 0.01.

Next, we set out to confirm the candidates eliciting the strongest effects in our knockdown screen. We stably expressed the respective EGFP-tagged proteins, including truncated proteins and point-mutants in HeLa-Kyoto cells and compared DiD transfer between those cells to control cells expressing only the EGFP-tag (Fig. 4).

EGFP-Myo10-tail significantly reduced codeIT, when expressed in both donors and acceptors or only in the donors, but not when expressed in the acceptor cells alone (Fig. 4A). EGFP-Cdc42-wild-type did not affect codeIT in either donors or acceptors or both. EGFP-Cdc42-G15A (nucleotide-free *i.e.* inactive mutant) and -Q61L (GTP-locked *i.e.* constitutively active mutant) significantly reduced codeIT, when both donors and acceptors expressed them, and when only donors expressed them. When expressed in the acceptors alone, no significant effect was measured (Fig. 4B). Neither EGFP-Rab11a-wild-type, nor -Q70L (GTP-locked *i.e.* constitutively active mutant) expression in both donors and acceptors decreased codeIT significantly; but -S25N (GDP-locked *i.e.* dominant negative mutant) caused a strong and significant reduction (Fig. 4C). Expressing -S25N only in donors or acceptors, respectively, also decreased codeIT, but not significantly. The results for -S25N are consistent with a synergistic effect of expression in donors and acceptors. Stably expressed EGFP-Rab35-S22N (GDP-locked i.e. dominant negative mutant) moderately reduced codeIT, while the wild-type and –Q67L (GTP-locked *i.e.* constitutively active mutant) had no significant effects when expressed in both donor and acceptor cells (Fig. 4D). For example images see Supplementary Fig. S2 online.

To identify the transferred membrane compartment, we measured the transfer of a number of EGFP-tagged endosomal and plasma membrane marker proteins (Table 1, Supplementary Fig. S3 online). Of those, EGFP-myosin-Vc-full length (Myo5c), EGFP-Myo10-tail, EGFP-Myo10-3xPH, EGFP-Rab5a, EGFP-Rab7a, EGFP-Rab8a, EGFP-Rab11a, farnesylated EGFP (farnesyl-EGFP, the C-terminal domain of K-Ras fused to EGFP) and GPI-anchored EGFP (EGFP-GPI) transferred significantly more than EGFP alone. EGFP-Rab35, the EGFP-tagged PH-domain of phospholipase Cδ (PLCδ-PH), which binds specifically to PI(4,5)P2, transferred even stronger. EGFP-Myo10-full length and heavy mero-Myo10 (Myo10-HMM), which contains the head and neck domains of Myo10, transferred the most. Contact-dependency of the transfer of EGFP-Myo10-HMM was confirmed by the same micro-patterning approach that has been described above (Supplementary Fig. S4 online).

Among the validated candidates affecting codeIT, only EGFP-Cdc42 did not transfer itself. We also could not detect transfer of the EGFP-EEA1-C-terminus (EEA1-CT), containing the PI3P-binding FYVE-domain, nor of a number of additional Rabs: EGFP-Rab1a, EGFP-Rab9a and EGFP-Rab7b, the trans-membrane proteins EGFP-E- and EGFP-N-Cadherin, and EGFP-Myo5c-tail. For the EGFP-2xFYVE construct, transfer was significantly higher than EGFP alone, but background levels were equally elevated (Table 1). For expression levels of all transfected constructs see Supplementary Table S2 online.

Parallel measurement of codeIT of DiD revealed that, of all constructs tested, only transient expression of EGFP-Myo10-HMM, EGFP-Rab8a-wild-type and EGFP-Rab8a-Q67L significantly increased codeIT of DiD over background (Supplementary Table S3 online). In a related study, we also found that overexpression of EGFP-Rab8a-Q67L (GTP-locked *i.e.* constitutively active mutant) in HeLa cells increased the number of transferrin receptor positive nanotubes as well as the transfer of transferrin receptor (Anne Burtey, personal communication). Expression of EGFP-2xFYVE significantly increased both codeIT of DiD and background. No construct reduced codeIT of DiD with significance.

### Inhibiting transfer of H-Ras

**Figure 5:**
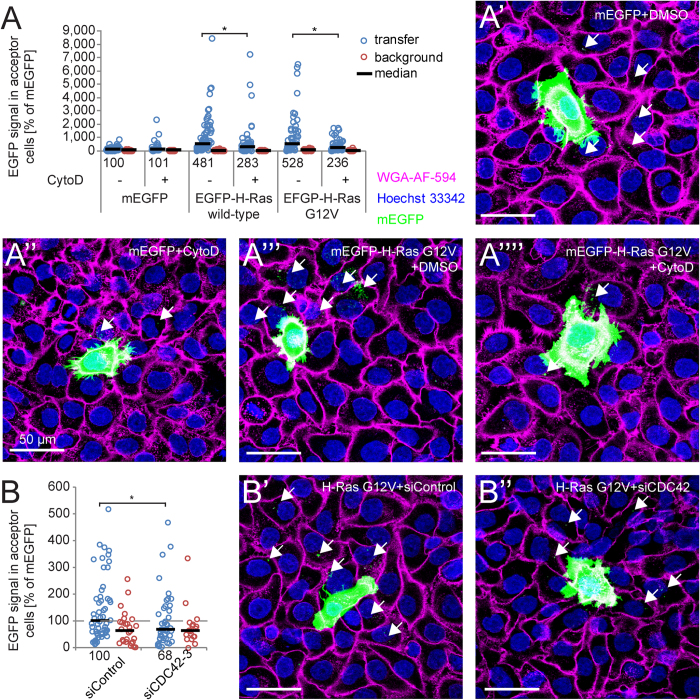
Intercellular transfer of oncogenic H-Ras is dependent on F-actin and Cdc42. Cells were transfected with constructs coding for mEGFP, mEGFP-H-Ras or mEGFP-H-Ras-G12V and co-cultured, stained, fixed, imaged and analysed according to methods. Circles show EGFP intensity in acceptor cells (transfer or background). (**A**) Co-cultures were treated with 100 nM CytoD in DMSO (+) or DMSO as control (−) for 20 h, beginning 2 h after plating of the co-cultures. Transfer of both wild-type and oncogenic mEGFP-H-Ras was significantly reduced, while transfer of mEGFP alone was unaffected. (**B**) Cells were transfected with mEGFP-H-Ras-G12V. After 24 h, transfected cells and non-transfected cells were separately cultured on siRNA-coated culture plates for 24 h and co-cultured again on siRNA-coated plates for 22 h, fixed, stained, imaged and analysed. Transfer was quantified as described above. SiRNA targeted against *CDC42* (si*CDC42*-3) significantly reduced transfer of oncogenic H-Ras. *ANOVA, followed by *post-hoc* Dunnett’s test p < 0.01. Scale bars 50 μm.

Since it had been shown that intercellular transfer of oncogenic H-Ras can affect immune cells^14^, we applied our approach to measure codeIT. In our assay, H-Ras wild-type and oncogenic mutant H-Ras-G12V (GTP-locked i.e. constitutively active), both tagged with monomeric EGFP (mEGFP), transferred significantly more than mEGFP alone (Fig. 5A–A”). Transfer of both proteins could be inhibited significantly by a low concentration of CytoD, while mEGFP transfer and background levels were unaffected (Fig. 5 A–A””). Knockdown of CDC42 by specific siRNA also significantly reduced transfer of oncogenic H-Ras compared to control siRNA (Fig. 5 B–B”).

### Automation of image acquisition

In order to facilitate large scale high-throughput screening formats, we automated the image acquisition using Leica MatrixScreener® and an in-house pattern-recognition program written in MATLAB (DonorFind). First, using a matrix of the part of the 24-well plate, which is within the scan range of the microscope (Supplementary Fig. S4A online), an autofocus map is acquired. Second, a low-resolution scan of Hoechst and DiD channels covering entire wells in 2D is acquired. Individual images are assembled into super-images spanning the entire well. Then, regions of interest identifying either single donor cells or no donor cells at all are determined with DonorFind. The coordinates of these regions are sent back to the microscope, and higher resolution 3D stacks in Hoechst, WGA, EGFP and DiD channels are acquired at the specified position (Supplementary Fig. S5B online). As proof of principle, we re-performed the CytoD inhibition experiment, achieving results in very good accordance to the standard method (Supplementary Fig. S5C online).

## Discussion

We present here a novel, microscopy-based method for automated quantification of codeIT. In our low-ratio confluent cell culture approach, codeIT is increased due to the larger contact area between cells, compared to commonly employed low-density approaches. Using a very low donor to acceptor cell ratio, the contribution of transfer through the medium was minimised and codeIT could be assigned to specific donor cells. Cells in image stacks were segmented by CellSegm^34^ in 3D. This enabled donor cell recognition and allowed integration of transfer intensity for each individual cell by our newly developed software TransQuant.

The presented method is superior to quantification of intercellular transfer by flow cytometry in several aspects. It 1) excludes signal resulting from the uptake of apoptotic cells, 2) differentiates between signals from inside and outside acceptor cells, 3) assigns transfer events to specific donor cells and 4) monitors cell morphology, expression levels, spatial arrangement of cells and parameters of transferred material such as density, distribution and size. Automatic quantification allows for higher throughput and accuracy. Furthermore, it is less biased and labour-intensive than manual evaluation of confocal images.

**Figure 6:**
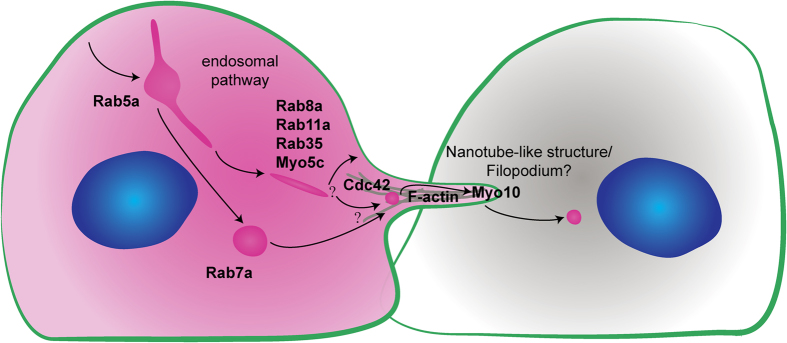
Model depicting published roles of candidate proteins and their presumptive involvement in codeIT in agreement with our results. We found codeIT to be influenced by the knockdown or overexpression of various Rab proteins described to be localised to early, recycling, and tubular endosomes. CodeIT was also dependent on F-actin, serum components and myosins. We therefore suggest the following working hypothesis for further investigation of the mechanism of codeIT: F-actin-rich protrusions, positive for Myo10 and regulated by Cdc42, mediate codeIT of membrane material delivered by the endosomal pathway and influenced by the depicted candidates.

We applied this method with a pilot siRNA screen to study the mechanism of codeIT. We suggest a model integrating our results, shown in Fig. 6. The observed transfer characteristics of DiD are consistent with vesicle transfer rather than diffusion of molecules along a continuous membrane bridge. This is supported by the correlation of integrated intensity (sum of greyscale units of voxels) and volume (sum of voxels) of the transfer, which equally correlated for all siRNAs tested and control, even though the volume of codeIT differed between conditions. Hence, the density of each individual “package” is not affected but rather the total number or volume of packages that are transferred. If transfer occurred by diffusion along a continuous membrane bridge, the average transfer signal (*e.g.* DiD) intensity per voxel, which is the density, should change when the total transfer signal intensity is altered by siRNA knockdown. This did not happen, and therefore our data indicates transfer in packages, which change their volume or number upon siRNA knockdown. Such an effect would arise from directly transferred vesicles (Fig. 3C,D). This is in accordance with our observation of transfer of endosomal marker proteins.

We measured the intercellular transfer of various fluorescently-tagged proteins to gain insight into the transferred compartment (Table 1). Several proteins associated with the PM did transfer, i.e. EGFP-Myo10-tail, EGFP-Myo10-3xPH domains (binding to PI-(3,4,5)-P3), EGFP-PLCδ-PH (binding to PI-(3,4)-P2, as does Myo6, a hit in our screen), EGFP-GPI, f-EGFP and EGFP-Rab35. In contrast, other proteins with at least partial PM-localisation did not transfer, *i.e.* EGFP-Cdc42-wild-type and -Q61L, EGFP-E-Cadherin and -N-Cadherin. Additionally, we detected transfer of proteins that predominantly localise to the early, recycling, tubular and late endosomes, *i.e.* EGFP-Rab5a, EGFP-Rab11a, EGFP-Rab8a, EGFP-Rab7a and EGFP-Myo5c, while marker proteins of other membrane compartments, such as Rab1a and Rab9a, did not transfer. In accordance with this, it has been reported recently that prion aggregates co-localise with markers of early, recycling and late endosomes inside nanotubes^36^. Since the transferred PM-associated proteins reportedly also localise to organelles, we hypothesize that the bulk of codeIT does not originate from the PM, but rather from intracellular membranes, likely one or several compartments of the endocytic pathway. In favour of this hypothesis, the bulk of the DiD signal in both donor and acceptor cells localises to the perinuclear region, while the PM stains weakly in comparison. A part of codeIT may also originate from specific domains of the PM, enriched in phosphatidyl inositol phosphate (PI-P)s, such as protrusion tips. Alternatively, a specific sorting or gating mechanism could exclude certain molecules from being transferred, and enrich for others.

Interestingly, EGFP-2xFYVE, a marker of both early and late endosomes, binding to PI3-P, transferred more than EGFP alone, but led also to increased EGFP signal in control regions. Additionally, EGFP-2xFYVE expression increased the DiD signal intensity in control areas. This indicates that a different, contact-independent mode of transfer, possibly involving secretion of exosomes, is increased by expression of EGFP-2xFYVE. Furthermore, this case shows that our system also detects increases in contact-independent transfer.

Several known regulators of organelle transfer were confirmed by quantification with our method. Among our tested candidates, Myo10-full length and -HMM (lacking the membrane binding tail) transferred themselves the most. Both localize specifically to the tips of cell protrusions such as filopodia and nanotube-like structures^37^,^38^, suggesting that protrusions promote codeIT, even in confluent cultures. The truncation mutant Myo10-tail, lacking the motor domain, was shown to inhibit Myo10 function in phagocytosis^39^. Notably, the Myo10-tail only inhibited codeIT when expressed in donors but not in acceptors (Fig. 4A). This demonstrates that our assay is able to differentiate if the candidate protein is involved in codeIT in the donor or the acceptor cell. It also shows that Myo10-mediated phagocytosis is probably not involved in codeIT. In FACS studies of comparatively low-density cultures of CAD cells expressing the same truncated Myo10 variants, Gousset *et al.* found that only Myo10-full length but not -HMM enhanced DiD transfer. They suggested that both tail and motor are needed for transfer^38^. However, using our method, both Myo10-full length and -HMM increased codeIT and were transferred themselves. Therefore, the tail domain might not be necessary for transfer, but needed to bridge longer distances by nanotubes as needed in a low-density culture setup. Alternatively, the differences might be cell-type specific.

Previously, mutant Cdc42 was shown to abrogate M-Sec-induced nanotube formation in HeLa cells^40^. In our assay, Cdc42 mutants inhibited codeIT specifically in donor cells (Fig. 4B), similar to Myo10-tail. Interestingly, both the GTP-locked Q61L active mutant and the non-nucleotide-binding G12A inactive mutant showed identical effects. This suggests that active cycling of Cdc42 between the inactive and active state is needed for the promotion of codeIT, likely via its ability to remodel F-actin and initiate the formation of protrusions^41^. While Cdc42-knockdown reduced codeIT, transfer of EGFP-Cdc42 itself was not detected. These findings point towards an upstream regulatory role for Cdc42 rather than direct involvement in the transfer mechanism. Cdc42 also acts upstream of Myo10 in promoting transfer of melanosomes from melanocytes to keratinocytes^42^. Additionally, the authors of a recent publication conclude that codeIT of mCherry between senescent cells is dependent on Cdc42-regulated actin-polymerization^43^.

We also identified specific Rab proteins as potential regulators of codeIT. Contrary to Myo10 and Cdc42 mutants, the observed effect of EGFP-Rab11a-S25N (inactive mutant) was much stronger when expressed in both donor and acceptor cells than when expressed unilaterally, consistent with a synergistic effect (Fig. 4C). We propose that Rab11a, a recycling endosome regulator^44^,^45^, could be synergistically involved in both donors and acceptors. One known role of Rab11a is the transport of cell-cell adhesion molecules to the PM^46^, which are possibly needed on both sides of the transfer site to establish tight contact. Another interesting screen hit is *VAMP4,* which has been shown to provide membrane material for a specific kind of fast growing protrusion^47^ and could in a similar way act in the formation of the protrusions mediating codeIT.

Rab35, a known marker of tubular endosomes^48^, transfers itself the most of all tested endocytic markers. Additionally, Rab35-S22N (inactive mutant) expression reduced codeIT significantly (Fig. 4D). Rab35 could be involved in the mechanism underlying codeIT in several alternative or combined ways. Rab35 has been shown to co-localise with both F-actin and fascin. It is involved in the regulation of actin remodelling by determining the sites where F-actin polymerization occurs^49^,^50^,^51^. Likely via this role and/or by the transport of Rho GTPases^52^, Rab35 initiates the formation of filopodia^52^,^53^,^54^,^55^,^56^, which are known precursors of nanotubes. Finally, Rab35 is also important for the trafficking of cadherins to the plasma membrane^57^, their stabilization in adherens junctions^58^ and thus possibly in the anchoring of nanotubes to target cells.

Intriguingly, knockdown of the early endosomal marker Rab5a reduced codeIT while knockdown of *EEA1*, a known Rab5a-antagonist^59^, enhanced it. Rab5 regulates several fusion events in the intracellular membrane systems and Rab5-positive compartments host hepatitis C virus^60^, *Listeria monocytogenes*^61^ and *Trypanosoma cruzi*^62^, known to spread directly from cell to cell, thereby evading the immune system. Myo10 has been implicated in the intercellular spreading of *Shigella flexneri*^63^. Additionally, HIV spreads faster from cell to cell via nanotubes than via the classical extracellular pathway *in vitro*^24^. This highlights a possible involvement of codeIT in unconventional spreading of intracellular pathogens. Insights into the codeIT mechanism could lead to novel treatments for infectious diseases. We also show the involvement of codeIT in oncogenic H-Ras transfer, an important target for anti-cancer drugs.

Our screening approach provides a basis for further investigations to elucidate the mechanism and physiological role of codeIT. This method is suited for any fluorescently labelled molecule or structure, including pathogens. The automated image acquisition and analysis may be adapted for high-throughput chemical and genetic screening approaches and drug testing.

## Material and Methods

### Material

CellTracker™ Green CMFDA (5-Chloromethylfluorescein Diacetate) (CTG), Vybrant® DiD Cell-Labelling Solution, wheat germ agglutinin Alexa Fluor^®^ (WGA-AF) 488 and 594 conjugate, were acquired from Invitrogen Detection Technologies. Hoechst 33342, was acquired from Sigma. 24-well glass-bottom microscopy plates were acquired from Greiner Bio-One GmbH). For a complete list of all tested siRNAs, their sequence and construct number see Supplementary Table S1 online.

### cDNA constructs

Transient transfections were performed by either using Lipofectamine 2000 or LTX, and Plus Reagent according to the manufacturer’s recommendations or via electroporation as described previously^64^. To obtain fluorescent E- and N-cadherin, respective full-length cDNAs were PCR-amplified out of hECad-pcDNA3 and hNCad-pcDNA1 (original plasmids kindly provided by Carien Niessen, University Hospital of Cologne, Germany). Primer pairs were: 5′-CTCGAGATGGGCCCTTGGAGCCGCAGCCTCTCG-3′/5-CCGCGGGTCGTCCTCGCCGCCTCCGTACATGTC-3′ and 5-CTCGAGATGTGCCGGATAGCGGGGCGCTG-3′/5′-CCGCGGGTCATCACCTCCACCATACATGTCAGCAAG-3′, respectively. PCR fragments were digested at introduced XhoI and SacII sites and subcloned into the XhoI and SacII sites of pEGFP-C1 (Clontech Laboratories, Palo Alto, CA, USA). Functionality of the constructs was validated by transient expression in wtCHO cells, which led to recruitment to cell-to-cell contacts of endogenous beta-Catenin, which is otherwise not detectable in CHO cells. DNA plasmids expressing EGFP-Myo10 and EGFP-Myo10-HMM, EGFP-Myo10-3PH and EGFP-Myo10-tail and EGFP-Myo5c, EGFP-Myo5c-tail^65^ were generously provided by Richard Cheney, University of North Carolina at Chapel Hill; USA. pEGFP-C1 and pEGFP-F (farnesylated EGFP) were purchased (Clontech Laboratories, Palo Alto, CA, USA). PH-domain of PLCδ has been published previously^66^. EGFP-GPI^67^ was kindly provided by Jaqueline Trotter (University of Mainz, Germany). Human EGFP-Cdc42, EGFP-Cdc42-S15A and EGFP-Cdc42-Q61L were generously provided by Keith Burridge (University of North Carolina, Chapel Hill, USA). EEA1-CT-EGFP^68^ and EGFP-2xFYVE^69^ have been published previously. EGFP-tagged Rab1a was a generous gift of Jaakko Saraste (University of Bergen, Norway). Constructs coding for wild-type and mutants of EGFP-tagged Rab5a, Rab7a, Rab8a, Rab11a and Rab35 were generously provided by Mitsonuri Fukuda (Tohoku University, Miyagi, Japan). EGFP-tagged variants of Rab7b and Rab9a were generous gifts of Oddmund Bakke (University of Oslo, Norway). mEGFP-C1, mEGFP-H-Ras and mEGFP-H-Ras-G12V were obtained from Addgene (plasmids 54759, 18662 and 18666).

### Cell Culture, Fixation, Staining and Co-culture

All experiments were performed with HeLa-Kyoto cells, cultured in DMEM supplemented with 10% FCS and 1% Penicillin/Streptomycin, if not indicated differently. All cells were maintained under humidified air supplemented with 5% CO_2_, at 37 °C. 24 h prior staining, cells were plated at a density of 35,000 cells/cm2. Attached cells were stained in culture medium with 5 μl/ml DiD (200 ng/ml) for 20 min. Plates were tilted 3 times at 5 time points during the incubation period. Thereafter, medium was exchanged to normal culture medium 4 times within 2 h. Then cells were washed twice with PBS and harvested by trypsinisation (0.25% trypsin/EDTA) for 10 min and washed through 5 ml of culture medium by centrifugation at 129 g. The supernatant was removed sparing 100 μl, 2 ml fresh culture medium were added and cells hence singularised by pressure-pipetting with Pasteur pipettes for 15 times. The co-cultures were plated on PLL-coated or siRNA-coated glass-bottom plates and incubated at 37 °C for 2 h before medium was exchanged to medium containing 2 mM dThymidin. Full medium containing 100 mM CytoD or DMSO, or medium without serum was added at this step if indicated, all containing 2 mM dThymidin. For experiments with transiently transfected donor cells, co-culture time after medium exchange was only 16 h due to the short expression window of Myo10-full length.

Co-cultures were washed with PBS, then fixed with 4% PFA/4% sucrose for 35 min, and hence quenched with NH_4_Cl (50 mM)/PBS, for 2 min. WGA-AF (488 or 594) for membrane staining and Hoechst 33342 for nuclear staining were added after fixation. Samples were imaged either during 30 to 120 minutes after addition of membrane stains, or fixed again with PFA/sucrose for 15 min, quenched with NH4Cl/PBS for 2 min in order to reduce diffusion of dye to intracellular membranes. Fixed and stained plates were then stored with 1 ml PBS/well at 4 °C and proved to be suitable for imaging and cell segmentation for at least 9 days. When stored for longer than 5 days 0.02% sodium azide was added.

### Fabrication of the microfluidic chip and micro-patterned surfaces, and cell seeding

Photolithography was performed in a MJB4 mask aligner to fabricate SU-8-patterned Si-masters^70^ for replica moulding in polydimethylsiloxane (PDMS). For the photolithography process, photo-emulsion or chromium masks were fabricated by GeSiM GmbH or Delta Mask B. V., respectively, based on the layout generated in LayoutEditor. The Si-master used for casting of microfluidic chips contained a positive relief (100 μm high) of a capillary network composed of three 300-μm wide channels (inlets) that gradually converged into a single 900 μm-wide analysis channel (outlet). Si-masters used for micro-contact printing contained a battery of rectangular islands of size 100 × 40 μm (L × W), 5 μm deep and pitch between patterns of 20 × 60 μm.

### Microfluidic chip

PDMS microfluidic chips were moulded in a MicCell^®^ casting station by pouring pre-mixed (10:1 ratio of base to curing agent) Sylgard 184 elastomer onto the Si-master, curing (65 °C/4 h), cleaning (3:2 isopropanol/ddH2O (v/v)) and placing the PDMS mould into contact with a glutaraldehyde-activated glass coverslip. The capillary network was primed (70% ethanol), washed (warm 0.01 M PBS) and incubated (10 μg/ml fibronectin/PBS; 1 h at 37 °C). Chips were humidified incubated overnight at 37 °C prior cell seeding. Custom-made reservoirs were plugged in the inlets of the microfluidic chip. Reservoirs and the outlet of the microfluidic chip were connected to PID-controlled syringe pumps. The microfluidic set-up was mounted in an Olympus IX70 microscope equipped with a motorized XY stage and a piezo z-stepper, a Polychrome V monochromator and an Andor DU-885 camera controlled by IQ 7.0. Imaging was performed at 37 °C, humidity and 5% CO2. For cell seeding, stained HeLa-Kyoto cells were suspended in 1% FCS (2 × 106 cells/ml) and injected in the reservoirs while having a uniform flow rate of 3 μl/min within the chip. CTG-stained cells were infused through contiguous inlets 1 and 2, and DiD-stained cells through inlet 3. After cell attachment, complete growth medium was infused at 30 μl/min to remove unattached cells and then maintained at 10 μl/min during 48 h. Imaging (16 bit) was carried out using a 40x/1.40 NA oil-immersion objective at 10 random XY positions along the interface of CTG and DiD-stained HeLa-Kyoto cells.

### Micro-contact printing

PDMS stamps for micro-contact printing were fabricated as described above. Cut stamps were activated (oxygen plasma/20 s/0.2 mbar). Activated stamps were inked with 50 μg/ml fibronectin for 30 min, rinsed (PBS, then Milli-Q water) and blown dry (N_2_). Inked stamps were placed into contact with glutaraldehyde-activated glass coverslips for 1 min. Stamps were peeled off and the coverslip was incubated for 30 min at RT. The stamped coverslip was adhered to a 8-well flexiPERM^®^ slide and non-stamped areas were passivated with 2% (w/v) bovine serum albumin in PBS for 1 h at room temperature. CTG and DiD-stained HeLa-Kyoto cells were suspended in complete growth medium and mixed as described before. The cell suspension was pipetted onto the micro-contact printed coverslip to a cell density of 30,000 cells/cm2 and incubated at 37 °C for 30 min to allow cell attachment on the stamped areas. Subsequently, unattached cells were removed by thorough washing with complete medium. Cells attached on micro-patterns were cultured and imaged as described above.

### siRNA-mediated knockdown

To investigate the transfer of DiD under gene knockdown, solid-phase reverse transfection of siRNA was performed using a modified protocol from a previous publication^71^. 24-well plastic culture plates and 24-well glass-bottom plates were coated with a gelatin based solution containing siRNAs and lipofectamine. Therefore, 0.2% gelatin (w/v) were dissolved in 0.45-μm-filtered H_2_O (solution 1), and 1.37 g sucrose in 10 ml OptiMEM (solution 2) and 7 μl Lipofectamine 2000 were mixed with 7 μl H_2_O (solution 3). 12 μl solution 2 were mixed with 14 μl solution 3 and 20 μl silencer–select siRNA (3 μM), incubated for 20 min at room temperature. Then 29 μl solution 1 was added. The mixture (75 μl) was diluted 1:50 in H_2_O in two steps, resulting in 3.75 ml volume. 300 μl of this solution were applied per well and dried in a MiVAc vacuum concentrator starting at 60 °C, then cooled with applied vacuum to 37 °C and stored in sealed boxes containing silica gel drying pearls as desiccant for future use.

### Imaging

Cells were imaged with a Leica confocal SP5 (LasAF version 2.7.3.9723) in the resonant scanner mode; zoom 1.7; pinhole airy1; 40 × 1.25NA oil immersion objective; 512 × 512 pixel; z-distance 1.01 μm, line-average 16; offset -1; gain 900–1,000 V. Images containing one donor cell and the surrounding acceptor cells were defined as ROI. For DiD- and EGFP-channels, laser power was adjusted to a strength resulting in saturated signal in approximately half the donor cell volume in order to be able to quantify the transferred DiD in the surrounding cells. For unknown reasons, some DiD stained cells displayed very weak staining, so that no saturated pixles occurred at these settings. ROIs with such cells were excluded. For all other channels the laser power was adjusted to prevent over-exposure.

### Automated image analysis

Cell segmentation and quantification of transfer: All algorithms for segmentation and automated analysis were written in MATLAB. The cell segmentation was done with CellSegm which was published previously^72^ and included the pre-processing step of anisotropic filtering, marker generation, watershed segmentation and classification of the watershed regions into background or cells. After watershed segmentation, the transfer of signal was analysed with TransQuant, which can be downloaded (https://github.com/dominikfr/Transquant). The algorithm automatically detects a DiD donor cell by high signal intensity. In absence of any donor cell, the stack is marked as a control stack. Small objects with a volume of less than 1,000 μm^3^ are discarded during donor cell definition. To fill all internal holes of the cell representations after thresholding, the images are then morphologically closed with a structural element of the two pixels. If both DiD and EGFP channels exist in a stack, the union of the cell representations from both channels is computed as approximation to the donor cell. The watershed segmented cell with the highest overlap to this approximation is then added to the donor cell as the union (with an OR operator). For a control condition where no donor cell is present, the watershed cell with the centre of mass closest to the image centre is used as donor cell. The detected donor cell is dilated with a structural element of radius (1,1,1; *i.e.* with one voxel in all directions) to ensure that there is no signal in the acceptor cells, which spatially belongs to the donor cell. Images for transfer analysis are defined as DiD or GFP channel. Signal above the global threshold (100 of 256 grayscale intensities for DiD, 100 or 160 of 256 for EGFP) was quantified as a read-out for transfer within the computed cell borders. The signal contained in non-donor cells was defined as intercellular transfer. Signal above the global threshold outside of cells was defined as unspecific transfer and was not counted as intracellular transfer. For signal quantification of transfer, the volume occupied by the donor cell definition is blanked (*i.e.* not counted). All watershed cells touching the boundary of the image and watershed cells with a volume below 100 μm^3^ were removed from the remaining analysis, assuming the low volume is an indication of dead cells. The transferred signal within each cell is measured in terms of total volume of binarised signal as well as integrated signal intensity as a sum of grey scale values within the binarised signal volume, *i.e.* within the same area. The numbers from the analysis program were exported to a text file, and then imported into Excel for statistical analysis.

### Statistical analysis

For each experiment we normalized the integrated fluorescent intensity of codeIT to the respective control condition (EGFP expressing or control siRNA transfected cells), which was set to 100%. Since the amount of codeIT originating from the different donor cells was not normal distributed (Fig. 2B), the data was transformed logarithmically (to the base e; *i.e.* the natural logarithm, *i.e.* using Euler’s number) for significance testing. This transformation yielded a normal distribution (Test for normality: Chi-square test, Fig. 2C). All statistical tests used to detect differences between experimental conditions were performed on this transformed data. For multiple comparisons, ANOVA was used to test if significant differences exist between the datasets, followed by *post-hoc* Dunnett’s test to identify the different dataset. For single comparisons, e.g. transfer compared to background in one respective condition, 2-sided Student’s t-test was used. A p-value lower than 0.05 was considered to be significant. To visualize differences between experimental conditions in dot plots, the untransformed data and the median is shown. The median of the control condition was always set to 100%.

### Automated image acquisition

We automatized the image acquisition on a Leica SP5 confocal laser scanning microscope with the help of Leica MatrixScreener software (version 3) in combination with our own DonorFind program (https://github.com/dominikfr/Transquant ). MatrixScreener and DonorFind communicate through a TCP/IP port and the CAM module from Leica. We produced a scanning template for a low resolution screen with a 20x objective (HCX PL APO CS 20.0 × 0.70NA DRY UV), speed 400 Hz, 1x zoom, covering the entire reachable well area for a 24 well plate. Format: 87*142 scan fields of 775 μm width each (Fig S5A). Into each field we placed autofocus points as regular grid with 3 scan fields distance, at least 2 scan fields distance from the well rim. Setup within the Matrix Screener: Matrix Screener; Single rectangular Matrix; Setup Template: start coordinates: X: 9,700, Y: 7,500, field distance: 775 μm, dz offset: 20, AF (autofocus) job: Z-Galvo; first channel only; analyse type: Intensity based method; excitation 405 nm, pinhole airy 2, capture range 60 μm, steps 30. Format 64 × 64 pixels. Low resolution job: Link low resolution scanning job to autofocus job; xyt; zoom 1; excitation: 405 nm, 561 nm, 633 nm. Format 512 × 512 pixels. High resolution job: Link to autofocus job. Zoom 3.4; pinhole airy 1, xyz; sequential scan; Excitation: a) 405 nm, 561nm, 633 nm. Stack, sequential scan: from 15–65 μm, 1 μm z-distance, format 512 × 512 pixels, line average 16. Waiting job: is an empty job without defined parameters that has to be set after each well. First, all wells are marked as low resolution job and one scan field after each horizontal line of wells is marked as a Waiting job. The autofocus map is started manually. After completion of the autofocus map, the low resolution job is started from MATLAB via DonorFind. The waiting job after each horizontal line of wells has the function as a trigger to start pattern analysis by DonorFind. DonorFind then finds all donor cells and excludes those where other donor cells are located within 200 μm surrounding distance. Further requirements were a cell density of at least 15 nuclei within a 200 μm radius. These donor cells are listed for high resolution imaging. Furthermore, if possible, it finds up to 15 random positions without any previously defined donor cell, but with corresponding distance requirements met, as for the donor cells, and without spatial overlap with any other chosen high-resolution field. DonorFind thereafter starts high resolution jobs at all calculated donor and control positions. After the high resolution job is accomplished for the first line of wells, the procedure is repeated for the next line of wells.

## Additional Information

**How to cite this article**: Frei, D. M. *et al.* Novel microscopy-based screening method reveals regulators of contact-dependent intercellular transfer. *Sci. Rep.* **5**, 12879; doi: 10.1038/srep12879 (2015).

## Supplementary Material

Supplementary Movie 1

Supplementary Movie 2

Supplementary Movie 3

Supplementary Information

## Figures and Tables

**Table 1:** Intercellular transfer of EGFP-tagged proteins.

EGFP-taggedconstructtransfected	Transfermedian ± SEM[% of EGFP]	n	Backgroundmedian ± SEM[% of EGFP]	n	Dunnett’stest[vs EGFP]	ANOVAgroup
Cdc42	82 ± 30	47	49 ± 71	30		A
Myo5c-tail	86 ± 36	68	82 ± 15	73		A
EEA1-CT	89 ± 28	49	88 ± 12	49		A
Rab9a	99 ± 9	51	109 ± 15	38		A
EGFP	100 ± 11	102	81 ± 6	55		A
Rab1a	103 ± 17	45	80 ± 15	44		A
E-Cadherin	113 ± 11	97	92 ± 14	30		A
N-Cadherin	123 ± 16	56	96 ± 26	20		A
Rab8a-T22N	126 ± 32	45	157 ± 19	35		A
Rab7b	148 ± 35	61	124 ± 16	39		A
2xFYVE	182 ± 22	52	178 ± 40	40	*,†	B
Myo10-3xPH	184 ± 48	28	101 ± 7	48	**	B
Rab11a	190 ± 45	85	130 ± 17	56	**	B
Rab5a	216 ± 72	28	124 ± 36	38	*	B
Rab8a-Q67L	227 ± 58	73	122 ± 22	45	**	B
Rab7a	235 ± 36	41	109 ± 14	41	**	B
Myo10-tail	250 ± 40	29	88 ± 10	20	**	B
farnesyl-EGFP	251 ± 48	64	96 ± 21	20	**	B
EGFP-GPI	276 ± 33	82	95 ± 26	15	**	B
Rab8a-wild type	315 ± 74	62	102 ± 51	40	**	B
Myo5c-full	331 ± 133	92	126 ± 19	74	**	B
Rab35	441 ± 89	84	53 ± 17	54	**	C
PLCδ-PH	447 ± 52	33	140 ± 21	42	**	C
Myo10-full	1,240 ± 630	16	103 ± 12	29	**	D
Myo10-HMM	4,015 ± 280	51	134 ± 15	13	**	E

Cells were transfected with plasmids coding for the indicated proteins. Transfected cells were co-cultured with non-transfected cells in a ratio of 1:400 for 22 h and then fixed, stained and imaged. Amounts of transferred proteins were measured as integrated EGFP fluorescence intensity in acceptor cells and compared to cells expressing the EGFP tag alone (set to 100%). Values are given as median ± standard error (SEM). n = number of image stacks quantified. ANOVA, followed by post-hoc Dunnett’s test were performed comparing the transfer of candidate proteins to EGFP and Student’s t-test was used for comparison to respective background. Proteins for which the signal was found to be significantly higher than both EGFP (ANOVA groups B-E) and background levels, were considered to be transferred. Significance levels for Dunnett’s test are indicated by *(p < 0.05) or **(p < 0.01). ^†^indicates that codeIT was not significantly higher than background. Proteins for which the signal was found to be not significantly higher than either EGFP (ANOVA groups A) or background levels were not considered to be transferred.
