# Erratum: Novel microscopy-based screening method reveals regulators of contact-dependent intercellular transfer

**DOI:** 10.1038/srep14158

**Published:** 2015-09-21

**Authors:** Dominik Michael Frei, Erlend Hodneland, Ivan Rios-Mondragon, Anne Burtey, Beate Neumann, Jutta Bulkescher, Julia Schölermann, Rainer Pepperkok, Hans-Hermann Gerdes, Tanja Kögel

Scientific Reports 5: Article number: 1287910.1038/srep12879; published online: 08142015; updated: 09212015

The Competing financial interests statement in this Article should read:

“The University of Bergen has pending patent applications on the screening technology. Family members of the European patent, Application No. 13194536.2 – 1408 are pending. The six authors T.K., H.-H.G., E.H., D.M.F., A.B. and I.R.M. are named as inventors in these applications”.

