# Supplementary material for: Novel microscopy-based screening method reveals regulators of contact-dependent intercellular transfer
Source: Sci Rep. 2015 Aug 14;5:12879. doi: 10.1038/srep12879 (PMC4536488; doi:10.1038/srep12879)
Supplement: Supplementary Information [file srep12879-s4.pdf]

Novel microscopy-based screening method reveals regulators of contact-dependent intercellular transfer

Dominik Michael Frei[1,2], Erlend Hodneland[1,3], Ivan Rios-Mondragon[1], Anne Burtey[1,4], Beate Neumann[5], Jutta Bulkescher[5,6], Julia Schölermann[1], Rainer Pepperkok[5], Hans-Hermann Gerdes[1,7] and Tanja Kögel[1,8,*]

[1]Department of Biomedicine, University of Bergen, Jonas Lies Vei 91, N-5009 Bergen, Norway; [2]present address: Department for Biosciences, Section for Physiology and Cell Biology, University of Oslo, Postboks 1066 Blindern, 0316 Oslo, Norway; [3]present address: Christian Michelsen Research, Fantoftvegen 38, 5072 Bergen, Norway; [4]present address: Department of Biosciences, Section for Biochemistry and Molecular Biology, University of Oslo, P.O. Box 1066 Blindern, 0316 Oslo, Norway [5]Advanced Light Microscopy Facility, European Laboratory of Molecular Biology (EMBL), Meyerhofstraße 1, 69117 Heidelberg, Germany; [6]present address: NNF Center for Protein Research, Protein Signaling Program, Blegdamsvej 3, 2200 Copenhagen, Denmark; [7]deceased august 18th 2013; [8]present address: National Institute of Nutrition and Seafood Research (NIFES), P.O. Box 2029, Nordnes, 5817 Bergen, Norway. *Correspondence should be addressed to T.K. (Tanja.Kogel@nifes.no).

Supplementary Figure 1

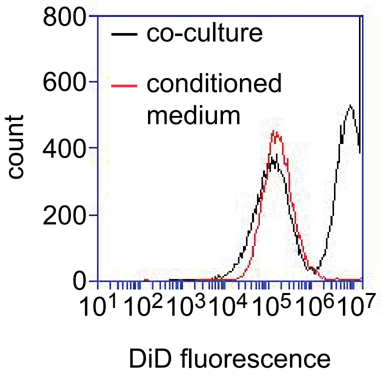

Representative example of DiD transfer analysis by flow cytometry in confluent 1:1 co-cultures. Cells were stained with DiD and co-cultured with cells stained with CTG for 24 h at indicated cell densities. DiD uptake was analysed by flow cytometry in 10,000 CTG stained cells for each cell density. The amount of cells with DiD transfer in confluent co-culture was compared to that of non-DiD stained cells of similar density, treated with medium conditioned by DiD-stained cells in a similar density. DiD transfer was analysed by flow cytometry. The number of cells with DiD transfer after co-culture and treatment with conditioned medium fluctuated between experiments, but was similar overall.

Supplementary Figure 2

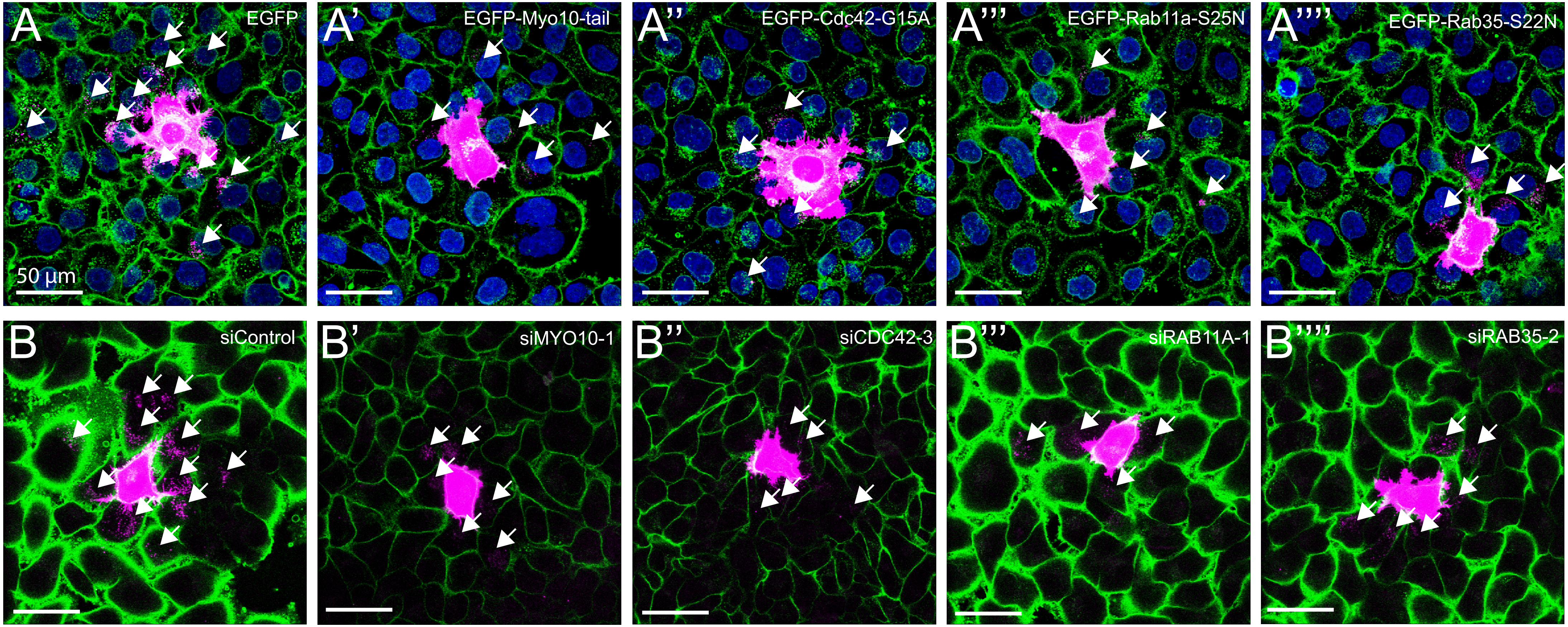

Representative example images for influence of protein expression and siRNA transfection on codeIT. (A-A'''') Images showing maximum projections after thresholding for DiD (magenta) and Hoechst (blue) channels and a single plane 2 µm above the substrate for WGA-AF-594 (green) of co-cultures with donor cells expressing the indicated proteins and acceptor cells stably expressing EGFP. (B-B'''') Images showing maximum projections after thresholding for DiD (magenta) and a single plane 2 µm above the substrate for WGA-AF-594 (green) of co-cultures transfected with indicated siRNAs. Scale bars, 50 µm.

Supplementary Figure 3

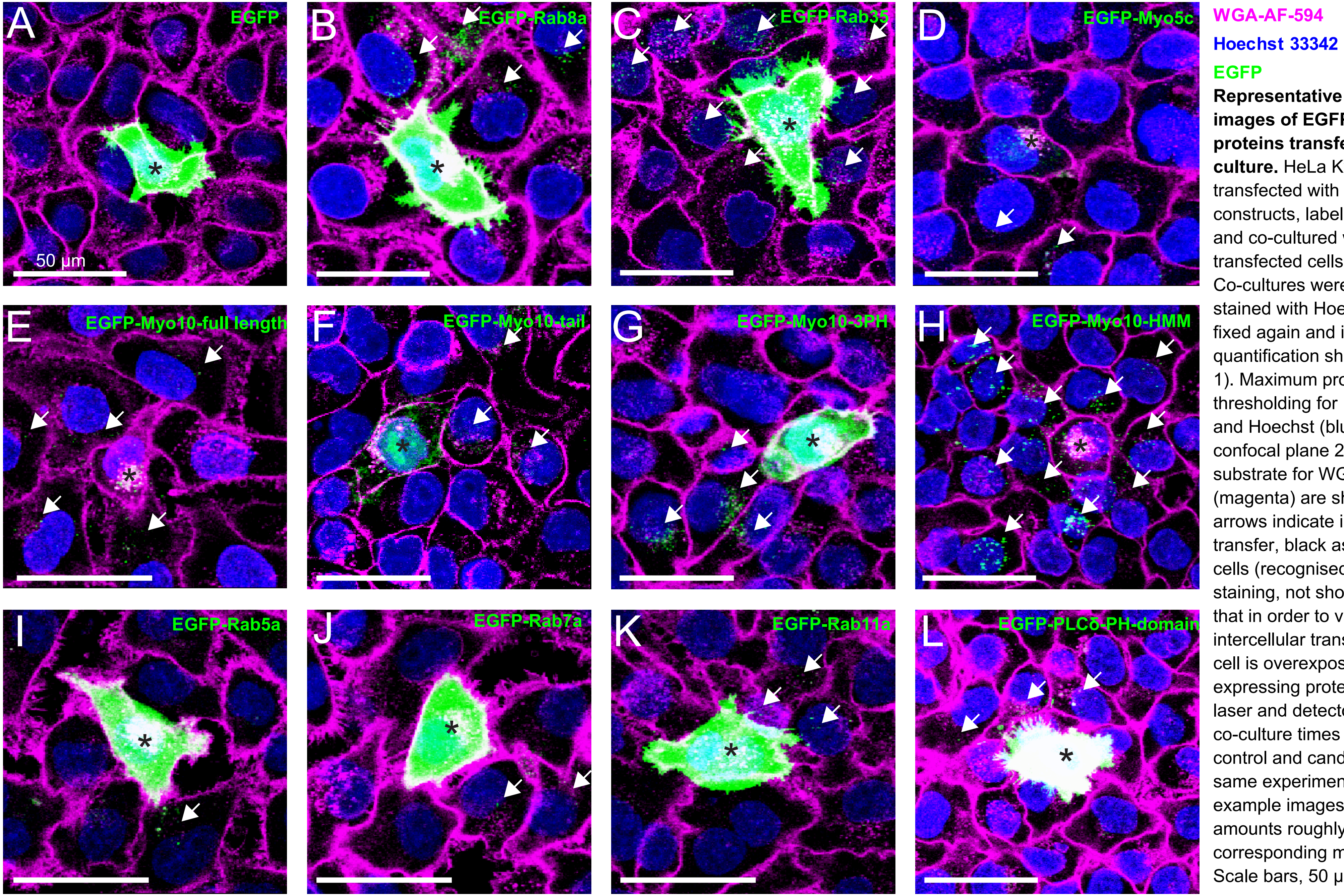

Representative example images of EGFP-tagged proteins transferring in co-culture. HeLa Kyoto cells were transfected with the indicated constructs, labelled with DiD and co-cultured with non-transfected cells for 18-22 h. Co-cultures were then fixed, stained with Hoechst and WGA, fixed again and imaged; quantification shown in (Table 1). Maximum projections after thresholding for EGFP (green) and Hoechst (blue), a single confocal plane 2 µm above the substrate for WGA-AF-594 (magenta) are shown. White arrows indicate intercellular transfer, black asterisks donor cells (recognised by DiD staining, not shown; *). Note that in order to visualize intercellular transfer the donor cell is overexposed for strongly expressing proteins (identical laser and detector settings and co-culture times were used for control and candidates in the same experiment). The chosen example images had codeIT amounts roughly equal to the corresponding median codeIT. Scale bars, 50 µm.

Supplementary Figure 4

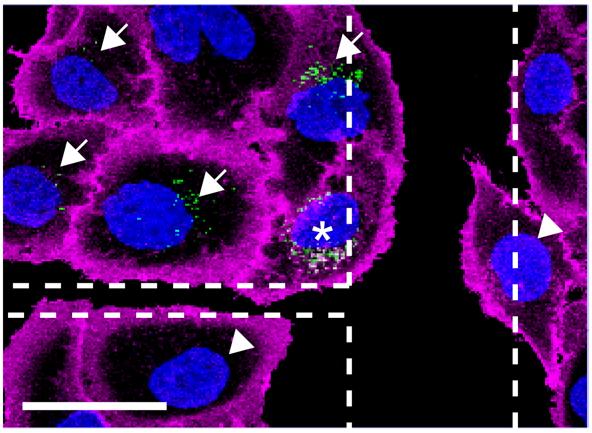

Intercellular transfer of EGFP-Myo10-HMM is contact-dependent. Cells were transfected with EGFP-Myo10-HMM, labelled with DiD and co-cultured with non-transfected, unstained cells at a ratio of 1:50. Cells were plated on a substrate containing stripes (outlined by white dashed lines) rendered non-adhesive for cells (see methods). Co-cultures were incubated for 18 h, then fixed, stained and imaged. Maximum projections for Hoechst (blue) and EGFP (green) channels and a single plane close to the substrate for WGA-AF-564 (magenta) are shown. Note that cells on the same side of the gaps receive transfer (arrows) from the donor cell (recognised by DiD staining, not shown; *), while cells separated by 60 μm and even 10 μm gaps (arrowheads) do not. Scale bar, 50 μm.

Supplementary Figure 5

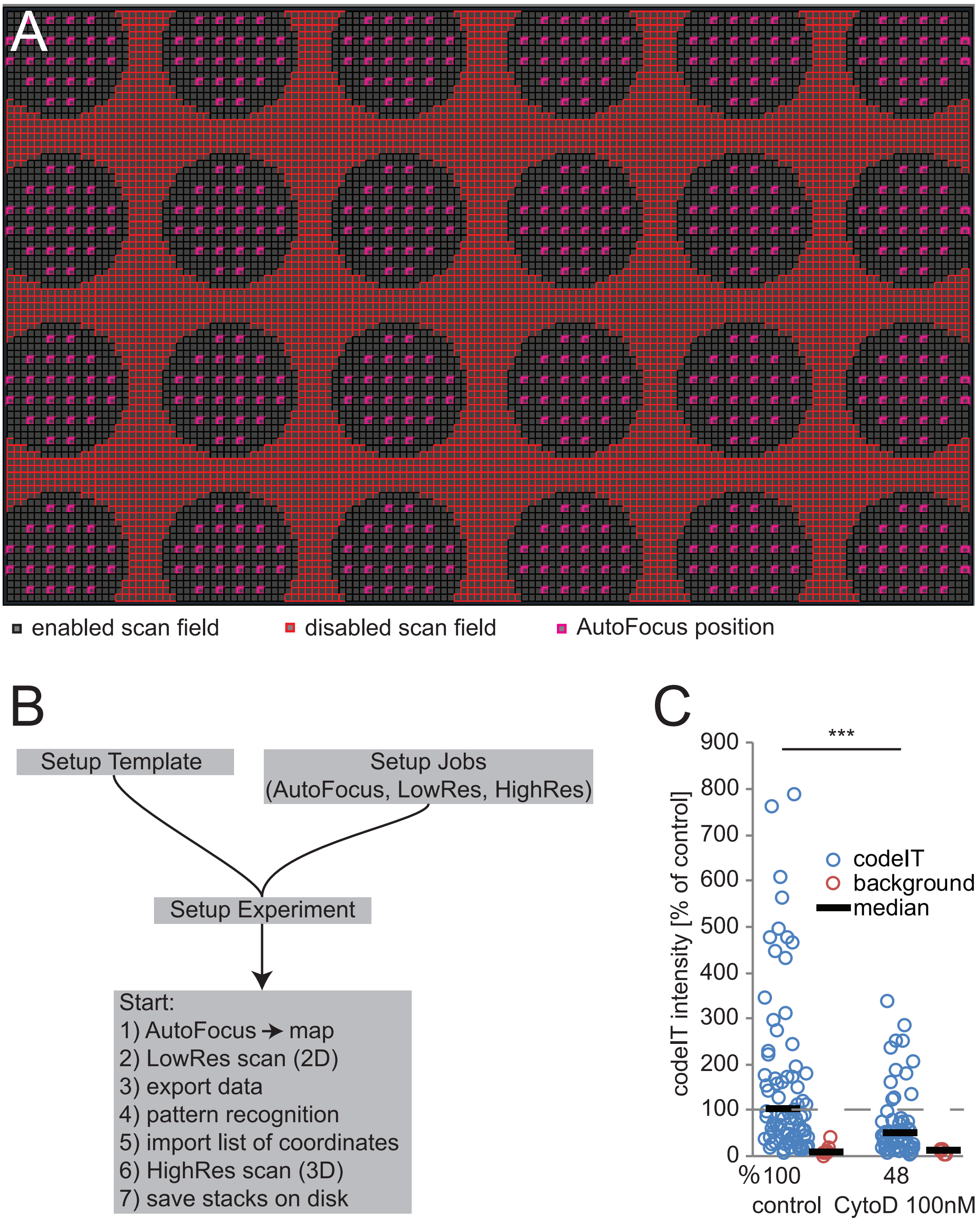

Automated confocal imaging. We automated the image acquisition on a Leica SP5 confocal microscope using Leica MatrixScreener® software and an in-house developed pattern recognition program (DonorFind), able to communicate with MatrixScreener via TCP/IP. (A) Schematic representation of the template assembled in MatrixScreener used for a low resolution scan of a 24-well plate. Note that the rim is limited by the scan range of the automatic xy-stage. Each field corresponds to one scan field of 775 x 775 µm size, thus covering the entire plate without gaps between scan fields. Grey fields, corresponding to *well* area in 24-well plates, are enabled, red fields are outside of wells and therefore disabled. Fields with a frame in magenta are positions for the AutoFocus. (B) Workflow diagram of automatic imaging using MatrixScreener. Every experiment consists of a template and imaging jobs, setup separately. First, an autofocus map of the imaging area is made and then a low-resolution map of the designated scanning area is acquired in 2D. DonorFind identifies regions of interest on this map corresponding to donor cells and control regions and creates a list of coordinates, which is sent back to MatrixScreener to acquire higher-resolution stacks at these positions. Stacks are saved on disk for subsequent analysis by CellSegm and TransQuant. (C) As a proof-of-principle, we repeated the experiment using 100 nM cytochalasin D to inhibit codeIT (compare to Fig. 1e). Blue circles indicate codeIT intensity, red circles background. *** $p < 0.001$, 2-sided Student's t-test; N = 56 for control and N = 76 for CytoD.

Supplementary Table 1

siRNA ID	Gene Symbol	Sense Sequence	Antisense Sequence	ENSG
**s1142**	AP1G1	GAUAUUCACCAGAACAUGAtt	UCAUGUUCUGGUGAAUAUCcg	ENSG00000166747
**s1143**	AP1G1	GGGAGGAAAUGACAUAACAtt	UGUUAUGUCAUUUCCUCCCaa	ENSG00000166747
**s1144**	AP1G1	GGUACGAAUUUUGCGGUUAtt	UAACCGCAAAAUUCGUACCtg	ENSG00000166747
**s17106**	AP3D1	GCCUAUGUCAGAUCAGUAUtt	AUACUGAUCUGACAUAGGCag	ENSG00000065000
**s17107**	AP3D1	ACAGGGCUCUGGAUAUUGAtt	UCAAUAUCCAGAGCCCUGUag	ENSG00000065000
**s17108**	AP3D1	CGCUGAAAAUUCCUAUGUUtt	AACAUAGGAAUUUUCAGCGag	ENSG00000065000
**s23817**	AP4E1	GUCUAUUCUAUUUAUCCUAtt	UAGGAUAAAUAGAAUAGACtg	ENSG00000081014
**s23818**	AP4E1	GAACUGCCCUUGGUUGAGAtt	UCUCAACCAAGGGCAGUUCtg	ENSG00000081014
**s23819**	AP4E1	CCUAAUCAAGUACAACAUAtt	UAUGUUGUACUUGAUUAGGag	ENSG00000081014
**s1565**	ARF6	GUCUCAUCUUCGUAGUGGAtt	UCCACUACGAAGAUGAGACct	ENSG00000165527
**s1566**	ARF6	AGACGGUGACUUACAAAAAtt	UUUUUGUAAGUCACCGUCUcc	ENSG00000165527
**s1567**	ARF6	CCAAGGUCUCAUCUUCGUAtt	UACGAAGAUGAGACCUUGGgt	ENSG00000165527
**s2765**	CDC42	UGGUGCUGUUGGUAAAACAtt	UGUUUUACCAACAGCACCAtc	ENSG00000070831
**s2766**	CDC42	UGAGAUAACUCACCACUGUtt	ACAGUGGUGAGUUAUCUCAgg	ENSG00000070831
**s2767**	CDC42	CAGUUAUGAUUGGUGGAGAtt	UCUCCACCAAUCAUAACUGtg	ENSG00000070831
**s2801**	CDH12	GGUACUUCAUAGAUUGGAAtt	UUCCAAUCUAUGAAGUACCta	ENSG00000154162
**s2802**	CDH12	CUCUCACCGAUGUCAAUGAtt	UCAUUGACAUCGGUGAGAGtg	ENSG00000154162
**s2803**	CDH12	GGAUUAGCCGGAACAACAAtt	UUGUUGUUCCGGCUAAUCCtc	ENSG00000154162
**s2771**	CDH2	GUGCAACAGUAUACGUUAAtt	UUAACGUAUACUGUUGCACtt	ENSG00000170558
**s2772**	CDH2	GGGUAAUCCUCCCAAAUCAtt	UGAUUUGGGAGGAUUACCCga	ENSG00000170558
**s2773**	CDH2	GAACAUAUGUGAUGACCGUtt	ACGGUCAUCACAUAUGUUCca	ENSG00000170558
**s4092**	DIAPH1	GGAGUUACGAUAGCCGGAAtt	UUCCGGCUAUCGUAACUCCca	ENSG00000131504
**s4093**	DIAPH1	GAUAUGAGAGUGCAACUAAtt	UUAGUUGCACUCUCAUAUCtt	ENSG00000131504
**s4094**	DIAPH1	GGACAUUCUUAAACGACUUtt	AAGUCGUUUAAGAAUGUCCaa	ENSG00000131504
**s4095**	DIAPH2	GAAGAUACUCAACGAAUUAtt	UAAUUCGUUGAGUAUCUUCtg	ENSG00000147202
**s4096**	DIAPH2	CGUCGAAAGCGGAUUCCAAtt	UUGGAAUCCGCUUUCGACGgt	ENSG00000147202
**s4097**	DIAPH2	GGACAUGAACCUUAACGAAtt	UUCGUUAAGGUUCAUGUCCtc	ENSG00000147202
**s15969**	EEA1	GCUAAGUUGCAUUCCGAAAtt	UUUCGGAAUGCAACUUAGCtt	ENSG00000102189
**s15970**	EEA1	GCUGGAUAAUACAACUGCAtt	UGCAGUUGUAUUAUCCAGCtt	ENSG00000102189
**s15971**	EEA1	GCAAUCUAGUCAACGGAGAtt	UCUCCGUUGACUAGAUUGCca	ENSG00000102189
**s4773**	EPS15	CAGCAUUCUUGUAAACGGAtt	UCCGUUUACAAGAAUGCUGtg	ENSG00000085832
**s4774**	EPS15	CUACCUUACUAGCCCAUAUtt	AUAUGGGCUAGUAAGGUAGaa	ENSG00000085832
**s4775**	EPS15	CAUUGUCAAAGGUCAACAAtt	UUGUUGACCUUUGACAAUGtg	ENSG00000085832
**s226733**	EVL	GGACAUCCAGAGAAGACAAtt	UUGUCUUCUCUGGAUGUCCat	ENSG00000196405
**s28180**	EVL	GAGUCGUUGGAGUCAAGUUtt	AACUUGACUCCAACGACUCtg	ENSG00000196405
**s28181**	EVL	GGCUUAAACUUUGCAAGUAtt	UACUUGCAAAGUUUAAGCCgt	ENSG00000196405
**s13207**	FSCN1	GAUCGACCGCGACACCAAAtt	UUUGGUGUCGCGGUCGAUCtc	ENSG00000075618
**s13208**	FSCN1	GCUGCUACUUUGACAUCGAtt	UCGAUGUCAAAGUAGCAGCtg	ENSG00000075618
**s13209**	FSCN1	GCAAGUUUGUGACCUCCAAtt	UUGGAGGUCACAAACUUGCca	ENSG00000075618
**s24494**	FSCN2	ACACCAUGUUUGAGAUGGAtt	UCCAUCUCAAACAUGGUGUtg	ENSG00000186765
**s24495**	FSCN2	CUACGUCUCUGUGCGGCAAtt	UUGCCGCACAGAGACGUAGcg	ENSG00000186765
**s24496**	FSCN2	GCCUCGUCAACGACACUGAtt	UCAGUGUCGUUGACGAGGCca	ENSG00000186765
**s26877**	FSCN3	GAGUGUAUCUGGGAAUUUUtt	AAAAUUCCCAGAUACACUCtc	ENSG00000106328
**s26878**	FSCN3	CGACCGCAUUCAUCUACUAtt	UAGUAGAUGAAUGCGGUCGgg	ENSG00000106328
**s26879**	FSCN3	CCUAAUCUCUGGUCGUUAUtt	AUAACGACCAGAGAUUAGGca	ENSG00000106328
**s9205**	MYO5A	GGAUAAGACGGUCCGUAAAtt	UUUACGGACCGUCUUAUCCtg	ENSG00000197535
**s9206**	MYO5A	CAGCCGUUUUGGGAAGUAUtt	AUACUUCCCAAAACGGCUGct	ENSG00000197535
**s9207**	MYO5A	GUAUAGUCCUAGUAGCUAUtt	AUAGCUACUAGGACUAUACca	ENSG00000197535
**s9208**	MYO5B	GAGACACGGUGUAUGAAGAtt	UCUUCAUACACCGUGUCUCtg	ENSG00000167306
**s9209**	MYO5B	CAUACACCAUGGAGGUAGAtt	UCUACCUCCAUGGUGUAUGtt	ENSG00000167306
**s9210**	MYO5B	CCAACCAUAUCUACACUUAtt	UAAGUGUAGAUAUGGUUGGac	ENSG00000167306
**s31792**	MYO5C	CGACGAUUCGUGCUUAAUAtt	UAUUAAGCACGAAUCGUCGga	ENSG00000128833
**s31793**	MYO5C	GAGUUGUCUUUCAAUCGGAtt	UCCGAUUGAAAGACAACUCtg	ENSG00000128833
**s31794**	MYO5C	CGAUUCGCAUUAGUGCACAtt	UGUGCACUAAUGCGAAUCGtt	ENSG00000128833
**s224086**	MYO6	GCACAAGGAUCAUUUUCGAtt	UCGAAAAUGAUCCUUGUGCtt	ENSG00000196586
**s9211**	MYO6	CCAAGAUGAUCUUCGAGUAtt	UACUCGAAGAUCAUCUUGGtc	ENSG00000196586
**s9212**	MYO6	GGAGCAAGCUUUAUUCGUUtt	AACGAAUAAAGCUUGCUCCag	ENSG00000196586
**s229976**	MYO7A	AGAUCCCUGUGAUGACCAAtt	UUGGUCAUCACAGGGAUCUtc	ENSG00000137474
**s9214**	MYO7A	GCGCGAAGAUUGAGCAGUAtt	UACUGCUCAAUCUUCGCGCcc	ENSG00000137474
**s9215**	MYO7A	GCACAAAGCUGAUCCUGCAtt	UGCAGGAUCAGCUUUGUGCtc	ENSG00000137474
**s9217**	MYO7B	AGAGCAUCCUUCUAGCCUAtt	UAGGCUAGAAGGAUGCUCUtt	ENSG00000169994
**s9218**	MYO7B	CAGACACCAUACUCCAUUAtt	UAAUGGAGUAUGGUGUCUGca	ENSG00000169994
**s9219**	MYO7B	GCCCAGAAGUUUAUAGACAtt	UGUCUAUAAACUUCUGGGCat	ENSG00000169994
**s9223**	MYO10	GAUAUUGACUGGAUAGACAtt	UGUCUAUCCAGUCAAUAUCtt	ENSG00000145555
**s9224**	MYO10	CGCUCUCUUCACUUACGAAtt	UUCGUAAGUGAAGAGAGCGta	ENSG00000145555
**s9225**	MYO10	GAUUUGACUUUAUCUACGAtt	UCGUAGAUAAAGUCAAAUCgg	ENSG00000145555
**s11675**	RAB4A	GGUCCGUGACGAGAAGUUAtt	UAACUUCUCGUCACGGACCtg	ENSG00000168118
**s11677**	RAB4A	CCUACAAUGCGCUUACUAAtt	UUAGUAAGCGCAUUGUAGGtt	ENSG00000168118
**s224510**	RAB4A	AGUUCUUGGUUAUUGGAAAtt	UUUCCAAUAACCAAGAACUta	ENSG00000168118
**s11678**	RAB5A	GGAAGAGGAGUAGACCUUAtt	UAAGGUCUACUCCUCUUCCtc	ENSG00000144566
**s11679**	RAB5A	CAAGCCUAGUGCUUCGUUUtt	AAACGAAGCACUAGGCUUGat	ENSG00000144566
**s11680**	RAB5A	GCAAGCAAGUCCUAACAUUtt	AAUGUUAGGACUUGCUUGCct	ENSG00000144566

**s15442**	RAB7A	GCUGCGUUCUGGUAUUUGAtt	UCAAAUACCAGAACGCAGCag	ENSG00000075785
**s15443**	RAB7A	GCUAGUCACAAUGCAGAUAtt	UAUCUGCAUUGUGACUAGCct	ENSG00000075785
**s15444**	RAB7A	GAGCUGACUUUCUGACCAAtt	UUGGUCAGAAAGUCAGCUCct	ENSG00000075785
**s8679**	RAB8A	GCAAGAGAAUUAAACUGCAtt	UGCAGUUUAAUUCUCUUGCca	ENSG00000167461
**s8680**	RAB8A	GAGUCAAAAUCACACCGGAtt	UCCGGUGUGAUUUUGACUCcc	ENSG00000167461
**s8681**	RAB8A	CUUUAAAAUUAGGACCAUAtt	UAUGGUCCUAAUUUUAAAGtc	ENSG00000167461
**s17916**	RAB9A	CCAGCUCUUCCAUACAAUAtt	UAUUGUAUGGAAGAGCUGGgt	ENSG00000123595
**s17917**	RAB9A	GGUCAGAUCAUUUGAUUCAtt	UGAAUCAAAUGAUCUGACCta	ENSG00000123595
**s17918**	RAB9A	GCUUCCAGAACUUAAGUAAtt	UUACUUAAGUUCUGGAAGCtt	ENSG00000123595
**s16702**	RAB11A	CAACAAUGUGGUUCCUAUUtt	AAUAGGAACCACAUUGUUGct	ENSG00000103769
**s16703**	RAB11A	GAGAUUUACCGCAUUGUUUtt	AAACAAUGCGGUAAAUCUCtg	ENSG00000103769
**s16704**	RAB11A	GGAGUAGAGUUUGCAACAAtt	UUGUUGCAAACUCUACUCCaa	ENSG00000103769
**s17647**	RAB11B	CUAACGUAGAGGAAGCAUUtt	AAUGCUUCCUCUACGUUAGtg	ENSG00000185236
**s17649**	RAB11B	GCAACGAGUUCAACCUGGAtt	UCCAGGUUGAACUCGUUGCgg	ENSG00000185236
**s225102**	RAB11B	GCAGAAAAGAACAACUUGUtt	ACAAGUUGUUCUUUUCUGCga	ENSG00000185236
**s32992**	RAB22A	UGAGCUACAUAAAUUCCUAtt	UAGGAAUUUAUGUAGCUCAtt	ENSG00000124209
**s32993**	RAB22A	CAGCUAUAAUCGUUUAUGAtt	UCAUAAACGAUUAUAGCUGca	ENSG00000124209
**s32994**	RAB22A	CGCCGACUCUAUUCAUGCAtt	UGCAUGAAUAGAGUCGGCGta	ENSG00000124209
**s21707**	RAB35	GCAGUUUACUGUUGCGUUUtt	AAACGCAACAGUAAACUGCtc	ENSG00000111737
**s21708**	RAB35	GAAGAGAUGUUCAACUGCAtt	UGCAGUUGAACAUCUCUUCca	ENSG00000111737
**s21709**	RAB35	GGCAUCCAGUUGUUCGAGAtt	UCUCGAACAACUGGAUGCCca	ENSG00000111737
**s19161**	SNAP91	GGAAUUUCUGGUACCACAAtt	UUGUGGUACCAGAAAUUCCaa	ENSG00000065609
**s19162**	SNAP91	CAAACGAUUUCUAACUAGAtt	UCUAGUUAGAAAUCGUUUGta	ENSG00000065609
**s19163**	SNAP91	GUUAUUAACUUACUCGAAAtt	UUUCGAGUAAGUUAAUAACac	ENSG00000065609
**s14439**	TSG101	GAAAAAGGGUCACCAGAAAtt	UUUCUGGUGACCCUUUUUCag	ENSG00000074319
**s14440**	TSG101	CUGUCAAUGUUAUUACUCUtt	AGAGUAAUAACAUUGACAGtt	ENSG00000074319
**s14441**	TSG101	GAGACCUAACUGUACGUGAtt	UCACGUACAGUUAGGUCUCtg	ENSG00000074319
**s16525**	VAMP4	CAAACAACUUCGAAGGCAAtt	UUGCCUUCGAAGUUGUUUGga	ENSG00000117533
**s16526**	VAMP4	GAUUUGGACCUAGAAAUGAtt	UCAUUUCUAGGUCCAAAUCtt	ENSG00000117533
**s16527**	VAMP4	AGCUUAUCGGAUAAUGCAAtt	UUGCAUUAUCCGAUAAGCUtt	ENSG00000117533
**s13670**	VAMP7	UCAUCAUCGUAUCAAUUGUtt	ACAAUUGAUACGAUGAUGAtg	ENSG00000124333
**s13671**	VAMP7	GACUACUUACGGUUCAAGAtt	UCUUGAACCGUAAGUAGUCtg	ENSG00000124333
**s13672**	VAMP7	GAUUGGAAUUAUUGAUUGAtt	UCAAUCAAUAAUUCCAAUCtt	ENSG00000124333
**s16522**	VAMP8	GGAGUUAAGAAUAUUAUGAtt	UCAUAAUAUUCUUAACUCCct	ENSG00000118640
**s16523**	VAMP8	UGAAGAUGAUUGUCCUUAUtt	AUAAGGACAAUCAUCUUCAcg	ENSG00000118640
**s16524**	VAMP8	GAACAUCUCCGCAACAAGAtt	UCUUGUUGCGGAGAUGUUCca	ENSG00000118640

siRNAs used in the screening. Internal siRNA ID, gene symbol, sense and antisense sequence, and Ensembl unique gene ID (ENSG) for all siRNAs used in the study. Upper case indicates matched and lower case unmatched bases.

Supplementary Table 2

EGFP-tagged construct transfected	expression median±SEM [% of EGFP]	n
N-Cadherin	14 ± 1	56
Myo10-full length	21 ±11	16
E-Cadherin	23 ± 2	97
Myo10-HMM	28 ± 5	51
Myo10-tail	29 ± 7	29
Myo5c-tail	49 ± 4	68
Rab5a	51 ± 6	28
Myo10-3xPH	53 ± 6	28
Rab8a-T22N	53 ± 6	45
Rab1a	63 ± 3	45
Rab7a	64 ± 5	41
2xFYVE	67 ± 3	52
EEA1-CT	69 ± 5	49
Rab8a-wild type	69 ± 6	62
Rab8a-Q67L	71 ± 4	73
Cdc42	73 ± 5	47
EGFP-GPI	78 ± 4	82
farnesyl-EGFP	78 ± 5	64
Rab9a	79 ± 2	51
Myo5c-FULL	80 ± 6	92
Rab7b	86 ± 3	61
PLCδ-PH	89 ±10	33
Rab11a	90 ± 3	85
Rab35	93 ± 3	84
EGFP	100 ± 3	102

Expression levels of EGFP-tagged proteins. Cells were transfected with plasmids coding for the indicated proteins. Transfected cells were co-cultured with non-transfected cells in a ratio of 1:400 for 18-22 h and then fixed, stained and imaged. Expression levels were measured as integrated EGFP fluorescence intensity in donor cells and compared to cells expressing the EGFP-tag alone (set to 100%). Values are given as median ± SEM. n = number of image stacks quantified.

Supplementary Table 3

EGFP-tagged construct transfected	DiD transfer median±SEM [% of EGFP]	n	background median±SEM [% of EGFP]	n	Dunnett's test [vs EGFP]
**Rab5a**	67 ± 15	27	15 ± 2	31	
**Cdc42**	70 ± 17	36	3 ± 4	30	
**Rab7a**	71 ± 10	40	19 ± 3	34	
**EEA1-CT**	80 ± 17	48	15 ± 14	49	
**Rab35**	91 ± 21	84	7 ± 5	54	
**Myo10-3xPH**	93 ± 34	29	14 ± 6	48	
**Rab7b**	94 ± 11	61	10 ± 6	39	
**f-EGFP**	96 ± 11	64	28 ± 2	20	
**Rab9a**	99 ± 40	51	8 ± 7	38	
**EGFP**	100 ± 11	71	9 ± 1	47	
**Rab11a**	100 ± 14	86	9 ± 9	56	
**Myo5c-tail**	105 ± 10	70	8 ± 4	73	
**PLCδ-PH**	106 ± 22	33	14 ± 6	42	
**Myo5c-full**	111 ± 14	91	11 ± 3	74	
**N-Cadherin**	115 ± 15	57	20 ± 2	20	
**2xFYVE**	117 ± 14	52	52 ± 25	40	
**Rab8a-T22N**	118 ± 16	45	7 ± 2	35	
**Myo10-tail**	126 ± 23	29	6 ± 1	20	
**Rab1a**	129 ± 25	46	6 ± 2	44	
**EGFP-GPI**	136 ± 13	82	16 ± 2	15	
**E-Cadherin**	147 ± 14	92	9 ± 2	30	
**Rab8a-wild type**	150 ± 15	56	12 ± 4	40	**
**Myo10-full**	184 ± 71	16	7 ± 1	29	
**Rab8a-Q67L**	190 ± 25	79	11 ± 2	45	**
**Myo10-HMM**	243 ± 32	51	4 ± 3	13	**

Effects on DiD transfer by expressing EGFP-tagged proteins. Cells were transfected with plasmids coding for the indicated proteins. Transfected cells were co-cultured with non-transfected cells in a ratio of 1:400 for 18-22 h and then fixed, stained and imaged. Amounts of transferred DiD were expressed as median integrated DiD fluorescence intensity in acceptor cells and compared to cells expressing the EGFP-tag alone (set to 100 %). Values are given as median ± SEM. n = number of image stacks quantified. ANOVA followed by *post-hoc* Dunnett's test were performed comparing the transfer of candidate proteins to EGFP alone. ** $p < 0.01$.

Movie descriptions

Movie 1 and Movie 2

Life-imaging in microfluidic chamber. DiD (magenta) and CTG (green) stained cells were plated into two adjacent areas using microfluidics. Subsequently cells were imaged by wide field microscopy every hour over a time period of 24 h, maintaining flow. Transfer exclusively occurs to cells that have contact to DiD labelled cells (yellow arrowheads, Movie 1). Note redistribution of transfer upon cell division (blue lines, Movie 2) Bundles of protrusions from DiD labelled cells orient dynamically towards the DiD accumulation of a recipient cell (white line). Scale bars, 50µm.

Movie 3

Confocal life-imaging captures transfer of DiD packages. DiD stained and unstained cells were co-cultured for 24 h and imaged life every second. Images represent a time lapse of maximum projections covering the entire height of the cell with 1 s time lapse between stacks. Blue arrowheads follow DiD organelles produced at protrusions of DiD labelled cells and released towards the bulk of transferred DiD in the recipient cell.
